# Under-ice convective regimes driven by sunlight and sediment temperature control water–ice heat flux

**DOI:** 10.1093/pnasnexus/pgag045

**Published:** 2026-03-04

**Authors:** Gustavo Estay, Daisuke Noto, Hugo N Ulloa

**Affiliations:** Department of Earth and Environmental Science, University of Pennsylvania, Philadelphia, PA 19104, USA; Department of Earth and Environmental Science, University of Pennsylvania, Philadelphia, PA 19104, USA; Faculty of Engineering, Hokkaido University, Sapporo 060-8628, Japan; Department of Earth and Environmental Science, University of Pennsylvania, Philadelphia, PA 19104, USA

**Keywords:** ice-covered waters, convection, solar radiation, sediment heat exchange, water–ice heat flux

## Abstract

Seasonal and perennial lake ice covers are declining, yet the mechanisms driving these changes remain insufficiently constrained. While atmospheric forcing is comparatively well understood, under-ice thermal dynamics—driven by sediment heat and solar radiation—are less certain. These energy inputs vary widely across lakes and seasons, and their contributions to ice melt are poorly quantified. We develop a minimal conceptual model that captures three essential elements of under-ice dynamics: the nonmonotonic equation of state, sediment–water heat exchange, and solar heating. Combining theory with direct numerical simulations informed by field observations, we map the under-ice dynamics, revealing five distinct regimes—one conductive and four convective—including three previously unrecognized. For each regime, we derive scaling laws linking governing parameters to the upward heat flux transferred to the ice. This framework provides a predictive foundation for quantifying under-ice heat transfer and parameterizing climate models, with implications for climate projections, biogeochemical cycling, and ecosystem dynamics in cryospheric waters.

Significance Statement Lake ice is melting faster and forming later each year. But predicting these changes remains a challenge for our limited knowledge on such seemingly quiet systems: what is happening underneath? We conceptualized ice-covered lakes mathematically to represent their unique features that rarely occur in tandem in other aquatic systems: isolation from the atmosphere by the ice-cover, sunlight absorption, heat exchange with sediments, and water density changes. Through detailed computational simulations, their interplays were found to host five distinct flow regimes beneath the ice cover—three previously uncharacterized. We modeled water–ice heat transport for all regimes, predicting ice warming. These findings not only shed light on under-ice dynamics but also provide insights into the evolution of the cryosphere in a warming climate.

## Introduction

The cryosphere is melting, with far-reaching impacts on climate and aquatic environments (1–4). Predicting these impacts requires a clear understanding of cold water systems. Ice-covered lakes, in particular, play a unique role by supporting ecosystems and critical services—including fishery, transportation, recreation, and energy production (5–8). Their dynamics are driven by two primary energy sources: sediment heat exchange and solar radiation that acts as an “internal heating” source. In early winter, when snow insulates the ice, the water beneath absorbs heat from the sediment—sourced from geothermal energy and summer heat storage—sustaining stratification or triggering deep convection (9–13). In late winter, once snow melts, sunlight penetrates the ice, directly heating the water and creating density gradients that can drive motion (14–21). A fraction of this absorbed energy is transferred to the ice, modulating its growth and melt (22–27). Yet, predicting this heat flux remains challenging, as sediment and solar heating vary widely and interact through coupled thermo-fluid processes, underscoring the need for a modeling framework that abstracts real-world complexity and quantifies each energy source’s role.

A defining feature of under-ice waters is their anomalous equation of state (EOS): between 0 ${\;}^{\circ }$C and ∼3.98 ${\;}^{\circ }$C density increases with temperature, so surface cooling promotes stratification rather than convection (28, 29). Yet, observations frequently reveal well-mixed layers—signature of convection. Convection driven by internal heating has been studied extensively, motivated by applications ranging from stellar interiors and Earth’s mantle to tidally heated planetary bodies (eg 30–38). By comparison, ice-covered waters have received less attention, and existing studies often emphasize case-specific configurations over general organizing dynamics (21, 39–46). In particular, these efforts largely overlook penetrative convection beneath stably stratified layers (47–52), a process expected to be especially relevant when bottom sediments retain summer heat or are warmed by groundwater and geothermal fluxes (9, 10, 13, 53–56). In nature, solar heating and sediment–water heat exchange act simultaneously, yet their coupled role in setting under-ice thermodynamics is still not resolved (28, 57). Here, we address this gap through a canonical conceptual model that isolates these processes and exposes their joint dynamical consequences.

Guided by field observations, we introduce two parameters—controlling internal solar heating and sediment–water heat exchange—to systematically explore the under-ice thermal dynamics by combining theory and direct numerical simulations. Our framework reveals five thermal regimes—four convective and one conductive—with distinct thermal and dynamical structure, and we elucidate the mechanisms governing transitions between regimes. We then derive closed-form scaling laws linking the governing parameters to the upward heat flux delivered to the ice cover. These results provide a predictive foundation for representing under-ice processes and ice-cover evolution in climate models, and for understanding their role as ecological hotspots in the cryosphere (5, 58–65).

## Results

### Conceptual model

To capture the fundamental physics of under-ice water bodies, our model is intentionally minimalist. We consider a liquid water layer of thickness *h* bounded above by a no-slip ice–water interface at a temperature of $\theta_{i}=0{\;}^{\circ }C$ (freezing point) and below by a no-slip sediment–water interface at a given temperature $\theta_{b}$, consequence of residual summer heat or geothermal heating. Lateral boundaries are periodic, eliminating edge effects. We represent the unique thermodynamics of shallow cold freshwater by using a quadratic EOS consistent with the temperature of maximum density $\theta_{r}\approx 3.98{\;}^{\circ }C$ (Fig. S1). There is ample evidence of ice-covered lakes with $\theta_{b}$ between $\theta_{i}$ and $\theta_{r}$ (16, 19, 66, 67), while cases with $\theta_{b}>\theta_{r}$ have also been recently reported in Tibetan lakes (23, 24, 66) and the American Great Lakes (53 , 55); in the latter, elevated sediment temperatures have been attributed to groundwater mean temperatures.

Radiative heating is modeled by the Beer–Lambert law (14), and controlled by the downward solar irradiance at the ice–water interface $E_{0}$, ie remaining radiation after reflection and direct absorption by the ice-cover (68). Reported values of $E_{0}$ spanning from 10 to 250 $W\;m^{-2}$ have been associated with convective flows in ice-covered lakes (eg 15 , 19, 69). Water opacity modulates the absorption of solar radiation and is quantified by the diffuse attenuation coefficient $K_{d}$ (28, 68). We parametrize the solar forcing through the radiative–conductive number $\mathrm{Rc}=E_{0}\;h/[k(\theta_{r}-\theta_{i})]$—where *k* is the thermal conductivity—and the dimensionless e-folding depth $\Lambda =1/(h\;K_{d})$, which compares the light-penetration length to the water body mean depth *h*. Here, we fix Λ=0.1, representing either a shallow, turbid system or a deep, clear one; this order of magnitude is observed in natural systems (14 , 28) and has been adopted in recent numerical studies (42, 45). To simplify the mathematical model, we work with a dimensionless temperature $\varphi =(\theta -\theta_{i})/(\theta_{r}-\theta_{i})$ (Supplementary material), taking the freezing point and the temperature of maximum density to be constants, $\varphi_{i}=0$ and $\varphi_{r}=1$. In this study, the bottom temperature $\varphi_{b}$ ranges in $0\leq \varphi_{b}<2$ and controls the sediment–water heat exchange. Thus, Rc and $\varphi_{b}$ capture the fundamental thermal forcings of under-ice water bodies.

Our model has two additional controlling parameters, the Prandtl number Pr≈11.67, which is fixed for the temperature range of interest (0 ${\;}^{\circ }$C–7.96 ${\;}^{\circ }$C), and the Grashof number Gr, the ratio between buoyancy and viscous force (see Mathematical formulation in Materials and methods).

Using this model, we combine theory with extensive 3D and 2D direct numerical simulations (DNS) to (i) examine how sunlight and sediment heat govern under-ice dynamics, (ii) delineate the resulting flow regimes, and (iii) quantify their impact on ice warming (Materials and methods and Supplementary material).

### Flow structures and regimes

**Fig. 1.**
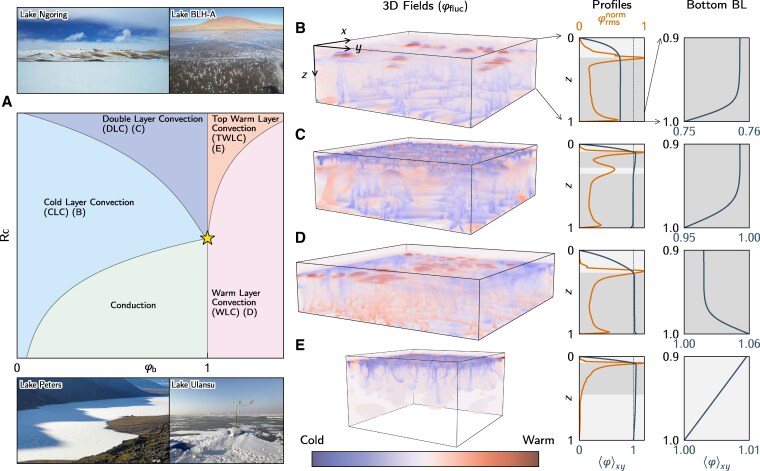
A) Phase diagram of under-ice dynamic regimes, with representative field images of ice-covered lakes showing temperature structures consistent with the identified thermal regimes: Lake Ulansu (70) (Credit: Prof Peng Lu and Puzhen Huo), Lake Peters (14) (Credit: Darrell Kaufman), Lake BLH-A (71) (Credit: Wang Huang), and Lake Ngoring (66) (Credit: Tom Shatwell and Giorgy Kirillin). B–E) Details of the four convective regimes: B) CLC (Movie S1, Rc≈9.35, $\varphi_{b}=0.75$), C) DLC (Movie S2, Rc≈18.71, $\varphi_{b}=0.95$), D) WLC (Movie S3, Rc≈5.61, $\varphi_{b}=1.06$), and E) TWLC (Movie S4, Rc≈18.71, $\varphi_{b}=1.0$)—including 3D snapshots of temperature fluctuations $\varphi_{\mathrm{fluc}}=\varphi -\langle \varphi \rangle_{xy}$, profiles of laterally averaged temperature $\langle \varphi \rangle_{xy}$, root-mean-square temperature fluctuations normalized by their maxima (for visualization purposes) $\varphi_{\mathrm{rms}}^{\mathrm{norm}}=(\langle \varphi_{\mathrm{fluc}}^{2}\rangle_{xy}{)}^{1/2}/\max\;(\langle \varphi_{\mathrm{fluc}}^{2}\rangle_{xy}{)}^{1/2}$, and close-ups of bottom BL. Shading highlights convective regions (dark) and stable regions (light).

Our modeling framework uncovers five under-ice dynamic regimes, one conductive and four convective, at steady state (Fig. 1).

The conductive regime is found by analytical integration of the governing equations (Materials and methods), without advection, under steady-state conditions. The system is quiescent and unconditionally stable—water density increase with depth—when the inequalities


(1) $$0\leq \varphi_{b}\leq 1\;\mathrm{and}\;\mathrm{Rc}\leq {\mathrm{Rc}}_{*}=\frac{\varphi_{b}}{\Lambda -\left(1+\Lambda \right)\exp\;\left(-1/\Lambda \right)}$$

are satisfied simultaneously. The bound ${\mathrm{Rc}}_{*}$ for the radiative–conductive number corresponds to the condition of zero heat flux at the sediment–water interface. The analytical solution of the temperature profile in this regime, denoted as $\varphi^{\mathrm{cond}}$, yields a close-form expression for the water-ice flux $\Phi_{i}^{\mathrm{cond}}=\partial \varphi^{\mathrm{cond}}/\partial z$ that depends only on prescribed parameters (Fig. S2). Outside the conductive regime, destabilizing density gradients energize the system to be unstable, requiring DNS to reveal their dynamics (Materials and methods).

To visualize 3D thermal structures, we use the temperature fluctuations with respect to the horizontally averaged temperature profile, $\varphi_{\mathrm{fluc}}=\varphi -{\left\langle \varphi \right\rangle }_{xy}$, as its sign indicates whether a water parcel is colder or warmer than the average temperature at its depth (Fig. 1, 3D fields). Together, the root-mean-square temperature fluctuations $\varphi_{\mathrm{rms}}=(\langle \varphi_{\mathrm{fluc}}^{2}\rangle_{xy}{)}^{1/2}$ signify horizontal variability; high values indicate regions of plumes’ formation or plumes’ impinging stable layers (Fig. 1, profiles).

The cold-layer convection (CLC) regime emerges at moderate Rc, while maintaining $\varphi_{b}<1$. The radiative heating allows the interior water to be warmer than the bottom, inducing unstable density gradients near the sediments (Fig. 1B, bottom BL). As a result, buoyant cold plumes rise from the bottom, sustaining convection throughout the water column, except in the vicinity of the ice cover (Fig. 1B, 3D fields, and Movie S1). This dynamics arises because the temperature of the entire system remains below 1, making warmer interior waters denser than the boundaries. The ice cover is isolated from the convective region by a stably stratified layer formed right beneath the surface. However, this layer is continuously deformed by the impinging of upwelling plumes, evidenced as a strong localized peak in $\varphi_{\mathrm{rms}}$ (Fig. 1B, profiles).

The double-layer convection (DLC) regime arises when, for $\varphi_{b}<1$, Rc is high enough to produce temperatures warmer than 1 in the upper region—where the absorption of solar radiation is prominent. As boundary temperatures are colder than 1, the temperature of maximum density is reached close to the ice, below a stable layer, producing sinking plumes that give shape to an upper convective region (Fig. 1B, 3D fields, and Movie S2). However, the temperature of maximum density is also achieved at an intermediate depth, with key ramifications for the flow structure. On the one hand, a stable density gradient is induced above this intermediate depth, preventing the aforementioned sinking plumes from reaching deeper regions. On the other hand, just below this depth, an unstable density gradient produces a new group of sinking plumes, which, together with cold plumes rising from the bottom (alike CLC regime), give shape to a lower convective layer. This bottom layer, with temperature below 1, is characterized by an S-shape temperature profile that resembles the paradigmatic Rayleigh–Bénard convection (RBC) (72).

When there is enough heat content, the warm water consolidates in an upper convective layer with a temperature above 1. In less energetic cases, the situation is slightly more complex, since the top warm water does not form a continuous layer. Instead, warm convective patches are localized in areas where the mixing from the lower convective layer is weaker (Fig. S9). In general, the warm and cold convective regions have different thicknesses and temperature gradients, explaining the distinct length scales and horizontal spacing of their plumes.

The warm layer convection (WLC) regime occurs in systems with $\varphi_{b}>1$ and low to moderate Rc. In this case, convection is driven by warm plumes rising from the sediment–water interface and cold plumes sinking from an intermediate depth where the water temperature reaches 1 (Fig. 1D, Movie S3). The temperature distribution of the convective layer resembles the S-shape profile of RBC. Above this layer, cold water forms a stable stratification that separates the convective region from the ice, alike penetrative convection observed elsewhere (30, 49, 51, 73, 74).

We identify the top warm layer convection (TWLC) regime occurring when $\varphi_{b}>1$ for high values of Rc. As a result of the strong radiative heating, the temperature of the convective layer exceeds the bottom boundary temperature $\varphi_{b}$, stabilizing the system from the bottom (Fig. 1E, profiles). As a result, convection is driven by sinking cold plumes formed near the ice cover, which then diffuse into the stably stratified layer below (Fig. 1E, 3D fields, Movie S4).

### Ice warming

**Fig. 2.**
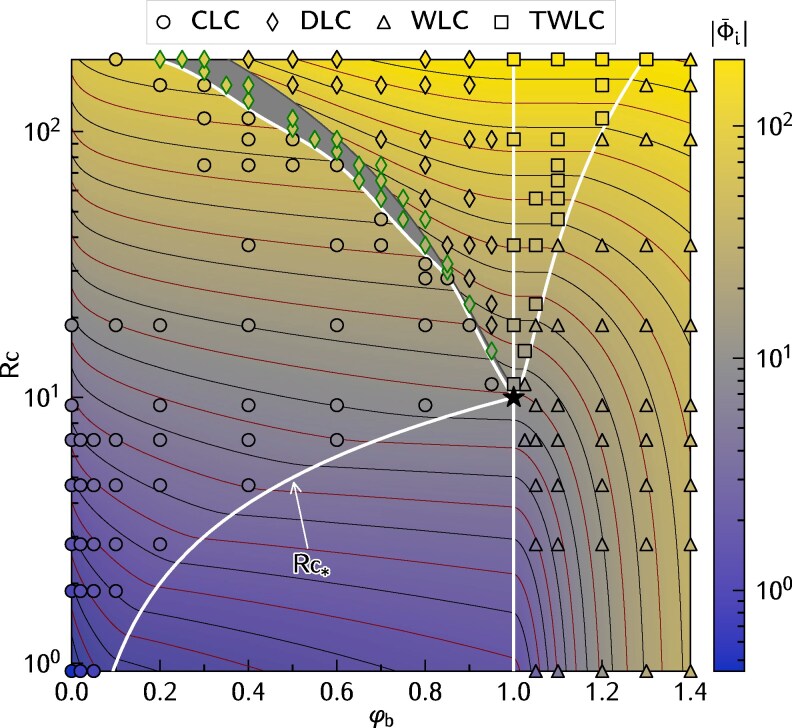
Regime diagram showing water–ice heat flux $\left|{\bar{\Phi }}_{i}\right|$ as a function of the radiative–conductive number $\mathrm{Rc}=E_{0}h/[k(\theta_{r}-\theta_{i})]$ and the dimensionless bottom temperature $\varphi_{b}$. Thick lines denote regime boundaries, symbols denote convective regimes. In particular, the boundary separating the conductive and CLC regimes is characterized by the function ${\mathrm{Rc}}_{*}(\varphi_{b})$ in Eq. 1. Scatter plots denote the results from direct numerical simulations and different symbols correspond to the convective regimes introduced in Fig. 1. The shaded area highlights a subregion within DLC regime, characterizing partial development of the upper convective layer. The green outlined diamonds represent cases with partially developed upper convective layer in the DLC regime. Thin contour lines denote iso-curves of constant water–ice heat flux $\left|{\bar{\Phi }}_{i}\right|$. The star symbol highlights the point where all regimes intersect.

We unveiled a gallery of convective regimes, realized by the interplay between solar radiation and sediment–water heat exchange (Fig. 2), with the aid of 2D DNS. Yet, a fundamental question remains: How do these convective dynamics influence ice warming? Despite their differences, all regimes share a key feature: a cold, stably stratified layer near the ice that modulates the heat transfer at the ice–water interface (Fig. 1, profiles). The heat flux at this interface, ${\bar{\Phi }}_{i}$, is always negative, meaning that liquid water loses heat and warms the ice. In contrast, the direction of the heat flux at the sediment–water interface is regime-dependent. In the conductive and WLC regimes, heat enters the system through the sediments (Fig. 1D, bottom BL and S2), while the opposite is true in the CLC, DLC, and TWLC regimes, as evidenced by the slope of their bottom boundary layers (BLs) (Fig. 1B, C, and E, bottom BL).

**Fig. 3.**
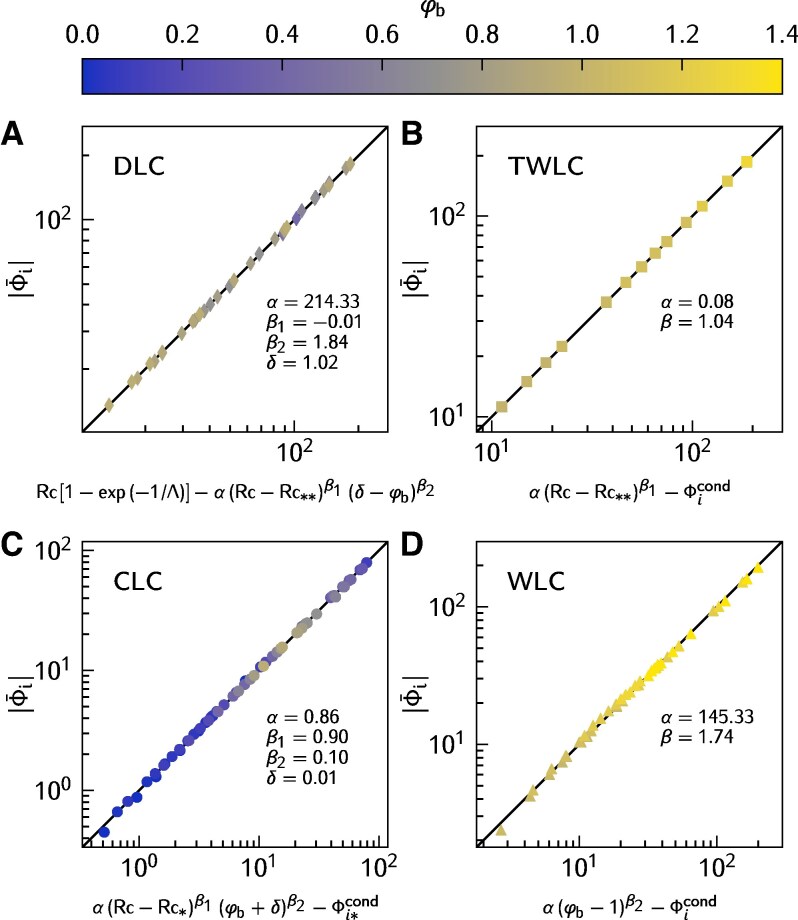
Fitting of scaling laws for water–ice heat flux $\left|\Phi_{i}\right|$ for each dynamic regime (Fig. 1). Comparison between measured $\left|\Phi_{i}\right|$ and the fitted scaling law for A) double layer convection (Eq. 4), B) top-warm layer convection (Eq. 6), C) cold layer convection (Eq. 3), and D) warm layer convection (Eq. 5).

We unearth the complexity of the upward heat flux at the ice–water interface $\left|{\bar{\Phi }}_{i}\right|$ in the four convective regimes (Fig. 2). For each regime, we fit a functional expression $\left|{\bar{\Phi }}_{i}|=f(\mathrm{Rc},\varphi_{b}\right)$ to our numerical results (Fig. 3), and complete the heatmap accordingly (Fig. 2). Note that the conductive regime allows us to derive an analytical expression of the heat flux. A general trend emerging from Fig. 2 is that $\left|{\bar{\Phi }}_{i}\right|$ increases with Rc and $\varphi_{b}$. Yet, each regime has its own particular behavior as we delve deeper next.

In the conductive regime, the behavior of $\left|{\bar{\Phi }}_{i}\right|$ is captured by the following analytical expression (Supplementary material):


(2) $${\bar{\Phi }}_{i}=\Phi_{i}^{\mathrm{cond}}=(\Lambda \;\mathrm{Rc})\left[1-\exp\;\left(-1/\Lambda \right)\right]-\mathrm{Rc}-\varphi_{b}.$$

From Eq. 2, and considering the adopted value Λ=0.1, the heat flux at the ice–water interface in this regime is bounded by a maximum value of $|{\bar{\Phi }}_{i}|\approx 10.0$.

In the CLC regime, we observe values of $\left|{\bar{\Phi }}_{i}\right|$ ranging from 0.4 to 79.3 in our dataset. To constrain the empirical model, we leverage the analytically determined conductive heat flux (Eqs. 1 and 2). Specifically, we consider the heat flux at the boundary between the conductive and CLC regimes $\Phi_{i*}^{\mathrm{cond}}=\Phi_{i}^{\mathrm{cond}}(\varphi_{b},{\mathrm{Rc}}_{*})$ as a reference, which depends on both $\varphi_{b}$ and *Λ*. Then, we propose


(3) $$f_{\mathrm{CLC}}=\alpha {\left(\mathrm{Rc}-{\mathrm{Rc}}_{*}\right)}^{\beta_{1}}{\left(\varphi_{b}+\delta \right)}^{\beta_{2}}-\Phi_{i*}^{\mathrm{cond}},$$

where *α*, $\beta_{1}$, $\beta_{2}$, and *δ* are fitting parameters whose values are reported in Fig. 3. We remark that $\beta_{1}$ is of order unity—making Rc almost linearly dependent with $f_{\mathrm{CLC}}$—with a correction factor that depends on $\varphi_{b}$ through the small exponent $\beta_{2}\approx 0.1$. We also note that $\varphi_{b}$ controls the heat flux linearly through the last term $\Phi_{i*}^{\mathrm{cond}}$. This formulation allows the model to identically match the analytical solution at the conductive–convective transition (Fig. 2).

As mentioned in the previous section, a crucial difference between the CLC and DLC regimes is that a portion of the water exceeds the temperature of maximum density in the latter, but not in the former. This criterion allows us to define their boundary in the parameter space. In the DLC regime, higher values of $\left|{\bar{\Phi }}_{i}\right|$ are observed, ranging from 13.5 to 182.8 in our dataset.

We employ a somewhat indirect approach to parametrize the water–ice flux in the DLC regime. The model is formulated from the perspective of the heat flux at the sediment–water interface ${\bar{\Phi }}_{b}$, leveraging the fact that density anomalies at this boundary are controlled by the known temperature difference “$1-\varphi_{b}$.” The water–ice flux is then linked to ${\bar{\Phi }}_{b}$ via the steady-state heat budget: $-{\bar{\Phi }}_{i}={\bar{\Phi }}_{b}+\mathrm{Rc}[1-\exp\;(-1/\Lambda )]$ (Fig. S5). Based on this, we propose the following model for the DLC regime:


(4) $$f_{\mathrm{DLC}}=\mathrm{Rc}\left[1-\exp\;\left(-1/\Lambda \right)\right]-\alpha {\left(\mathrm{Rc}-{\mathrm{Rc}}_{**}\right)}^{\beta_{1}}{\left(\delta -\varphi_{b}\right)}^{\beta_{2}},$$

where ${\mathrm{Rc}}_{**}$ serves as a reference value—obtained from the minimum radiative heating at which the DLC regime is observed. Here, the exponent $\beta_{1}$ is close to zero, so $(\mathrm{Rc}-{\mathrm{Rc}}_{**}{)}^{\beta_{1}}$ acts as a weak correction factor, whereas the dependence on $\varphi_{b}$ is nearly quadratic, with exponent $\beta_{2}\approx 1.84$. In particular, as $\varphi_{b}$ approaches 1, $f_{\mathrm{DLC}}$ is dominated by its linear dependence on Rc (first term in Eq. 4).

We note that the model in Eq. 4 does not apply within a narrow transitional zone of the DLC regime (shaded region in Fig. 2), where the behavior of the water–ice flux becomes notably complex. Part of this complexity is attributed to the aforementioned transition from patchy structures to a developed convective layer in the upper region. For consistency, we define the boundary between the transitional and fully developed DLC regime as the curve where the CLC (Eq. 3) and DLC (Eq. 4) models yield equal water–ice flux, ensuring a continuous representation of the heat flux across regime transitions.

In the WLC regime, $\left|{\bar{\Phi }}_{i}\right|$ shows the largest variability in our simulations, from 2.4 to 193.9. In analogy with RBC, in which the linear profile can be used as a reference for measuring the convective heat-flux, we employ the fully conductive water–ice flux $\Phi_{i}^{\mathrm{cond}}$ as a reference in our model for $\left|{\bar{\Phi }}_{i}\right|$:


(5) $$f_{\mathrm{WLC}}=\alpha {\left(\varphi_{b}-1\right)}^{\beta_{2}}-\Phi_{i}^{\mathrm{cond}}.$$

The boundary between the WLC and the TWLC regimes (Fig. 2) represents the condition of zero heat flux at the sediment–water interface. That is, internal heating provided by solar radiation is completely balanced by the heat flux at the ice–water interface: $\left|{\bar{\Phi }}_{i}|=\mathrm{Rc}[1-\exp\;(-1/\Lambda )\right]$ (Supplementary material).

In comparison to the DLC regime, the water–ice heat transfer in the TWLC regime is much less sensitive to the bottom temperature $\varphi_{b}$, being primarily controlled by Rc within the explored parameter space. This insensitivity stems from the isolation of the convective region from the bottom boundary due to the lower stable stratification. To parametrize the TWLC regime, the conductive heat flux $\Phi_{i}^{\mathrm{cond}}$ is again employed as a reference, leading to the following model:


(6) $$f_{\mathrm{TWLC}}=\alpha {\left(\mathrm{Rc}-{\mathrm{Rc}}_{**}\right)}^{\beta_{1}}-\Phi_{i}^{\mathrm{cond}}.$$

As in the DLC model (4), ${\mathrm{Rc}}_{**}$ defines the lower bound of the radiative forcing for the TWLC regime, anchoring the scaling to its minimum water–ice flux. This formulation reflects the passive role of the bottom temperature $\varphi_{b}$ in the enhancement of $\left|{\bar{\Phi }}_{i}\right|$ and the system’s strong sensitivity to radiative input.

These models show that the sensitivity of the heat flux with respect to the controlling parameters depends on the underlying thermal regime, which can be quantified by the partial derivatives $\partial |{\bar{\Phi }}_{i}|/\partial \mathrm{Rc}$ and $\partial |{\bar{\Phi }}_{i}|/\partial \varphi_{b}$ (Fig. S11). The sensitivity to $\varphi_{b}$ is lower in the conductive and TWLC regimes, and also in most of the CLC regime, with an exception for high values of Rc or values of $\varphi_{b}$ very close to 0. High sensitivity to $\varphi_{b}$ is observed in the DLC regime, but it decreases as $\varphi_{b}$ approaches 1. In contrast, the sensitivity increases with $\varphi_{b}$ in the WLC regime, eventually reaching very high values. The sensitivity to Rc varies less across the parameter space, with the exception of the CLC regime where it drops with the increase of Rc or the decrease of $\varphi_{b}$.

Remarkably, all regime boundaries intersect at a single point in the parameter space: $\varphi_{b}=1$ and $\mathrm{Rc}={\mathrm{Rc}}_{**}={\mathrm{Rc}}_{*}(\varphi_{b}=1)\approx 10.0$ (the star symbol in Figs. 1A and 2). From this locus, the system can transition into five distinct thermal and convective regimes, even with a slight variation of radiative forcing or bottom temperature. This convergence point—from where multiple dynamical pathways emerge—underscores the rich, complex, and somewhat perplexing behavior of under–ice convection, making it a genuinely intriguing system to explore.

## Discussion

Convective dynamics of ice-covered lakes emerge from a subtle interplay of nonlinear processes. Penetrative solar radiation, sediment–water heat exchange, and the nonmonotonic EOS of cold water collectively shape the vertical structure of the water column, hosting convective and stably stratified layers concomitantly. Our results reveal a spectrum of under-ice flow regimes that moves beyond the conventional dichotomy of “winter” lakes as either stably stratified (“Early Winter”) or single-layer convective systems (“Late Winter”) (29, 60), underscoring the need for a mechanistic exploration of ice-covered lakes’ circulation. Each regime exhibits unique dynamics and warrants targeted investigation to elucidate its implications for lake heat budgets, under-ice life, and climate sensitivity.

### Minimal conceptual framework

Our framework, which adopts the Beer–Lambert light absorption and a nonmonotonic EOS, might appear to be complex. But it is, in fact, minimal and cannot be simplified further without losing essential physics observed in ice-covered freshwater bodies. One might expect this framework to reduce to the classical system of internally heated convection with a spatially uniform heat source (33–35, 75) in certain limits. For example, for fully transparent ($K_{d}=0$) or fully opaque ($K_{d}\to \infty$) waters. However, both limits result in vanishing internal heating; in the former, light penetrates without absorption; in the latter, it is absorbed entirely at the surface. Therefore, a spatially homogeneous internal heat source cannot represent energy absorption from solar radiation. Another characteristic of this classical system, and similar ones that consider depth-dependent internal heating (76, 77), is that they are not concerned with the nonmonotonic EOS of water, predicting significantly different convective dynamics, both in flow structure and heat transfer laws (eg 33, 34). Moreover, recent studies have investigated convective dynamics in cold waters (78, 79), adopting a linearized EOS instead of a nonlinear model; the latter is well-justified when the temperature range is narrow (eg 0 ${\;}^{\circ }$C–1 ${\;}^{\circ }$C) (79, 80). Our study avoids such linearization, since it fails to accurately represent density gradients around the temperature of maximum density—crucial for the dynamics of the DLC, WLC, and TWLC regimes (Fig. 1). We emphasize that depth-dependent internal heating and a nonmonotonic EOS are fundamental to capturing the coexistence and spatial distribution of convective and stably stratified layers in ice-covered lakes.

The inherent nonlinear processes set by light absorption and EOS in ice-covered lakes make them challenging to scale. Even within our minimalist framework, five dimensionless parameters characterize the four convective regimes reported. This is a broad parameter space that will require subsequent studies to fully explore, as we have fixed the light penetration Λ=0.1 and the Grashof number $\mathrm{Gr}∼10^{8}$—whose influence on the convective regimes is discussed further in the Supplementary material. Moreover, additional physics present in natural systems further increases this complexity and merits future research. These include basin geometry (43, 46), lateral variability in solar irradiance (39, 40), salinity stratification (81, 82), Earth’s rotation (41, 43, 83, 84), and ice cover evolution (85). Such additional physics will likely enrich and potentially expand the gallery of under-ice convective regimes.

### Transient dynamics

Although our study focused on characterizing steady-state conditions—enabling the development of robust scaling laws that link governing parameters to the water–ice heat flux—the framework can simulate transient dynamics observed in natural systems. In seasonally ice-covered lakes, “early winter” is typically marked by snow accumulation over the ice, producing minimal light or complete darkness beneath the surface (ie Rc≈0) (28, 29). During this phase, sediment heat release is the primary energy source, warming deep waters while near-ice layers remain near freezing. If bottom temperatures stay below 3.98 ${\;}^{\circ }$C, the water column becomes stably stratified, evolving toward a statistically conductive regime with minimal kinetic energy (9, 11, 12). This thermodynamic state is widely documented in northern lakes (29, 60, 68).

As snow cover recedes and solar radiation penetrates the ice (Rc>0) in “late winter,” stratified waters destabilize, leading to the formation of a growing, well-mixed layer with nearly uniform temperature—an evolution captured in many ice-covered lakes, including Babine Lake, Lake Vendyurkoe, and Lake Onego (15, 16, 18). Although such a vertically uniform temperature region suggests active convection, few studies have confirmed it through simultaneous temperature and velocity measurements (15, 69, 86).

**Fig. 4.**
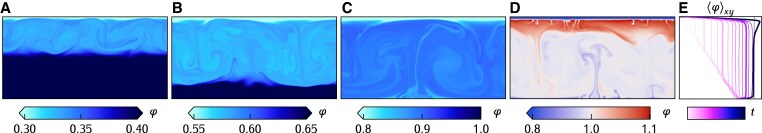
Transient dynamics in an ice-covered water body with bottom temperature $\varphi_{b}=0.8$. The system starts from rest and is forced by both solar radiation (Rc=46.8) and heat exchange at the sediment–water interface. A–D) Snapshots of the temperature field φ in an interior region, highlighting different stages in the development of a convective profile. E) The temporal evolution of laterally averaged temperature profiles, $\langle \varphi \rangle_{xy}$, over the full horizontal domain. Profiles with bold lines correspond to the instants shown in (A)–(D), respectively (Movie S5).

We challenge our framework to reproduce this transient process observed from early to late winter (Fig. 4). For this, we initialize a quiescent system with a linear temperature distribution between the ice–water and sediment–water interfaces (0<φ<1), and numerically integrate the equations of motion (Materials and methods) adopting typical parameter values obtained from the literature (Rc≈46.8, $\varphi_{b}=0.8$). A convective layer emerges beneath the conductive ice boundary and progressively deepens as it entrains the underlying stratified fluid. Eventually, the water column fully mixes, forming a column-scale, unstable convective layer with a temperature higher than the sediments—consistent with the CLC regime observed in field studies during “late winter.” Classic examples of the CLC regime are reported from Lake Peter and Babine Lake (14, 16). We remark that time-varying solar input is omitted here, though such forcing is readily implemented (43, 44, 46). Still, our results reproduce temperature distributions observed under diurnally varying Rc, where daytime solar heating drives rapid mixing, while nighttime diffusion is too slow to re-establish stratification—highlighting the primacy of sunlight forcing in driving under-ice convection.

In our transient simulation, continued solar heating eventually warms the upper region above φ>1, transitioning the system to a DLC regime (Fig. 1): one convective layer near the ice that accumulates heat, and a second convective layer persists below. While field studies have reported analogous temperature profiles—for instance in Lake Peters and the Tibetan Lake BLH-A (19, 23)—the existence of two distinct convective layers remains unconfirmed by direct velocity measurements.

### Heat collectors

Recent observations of Tibetan lakes—termed heat collectors—have expanded the known spectrum of thermo-fluid regimes in ice-covered lakes. Observations report that at some point the bottom waters exceed the temperature of maximum density (3.98 ${\;}^{\circ }$C), producing sediment–water heat fluxes that, in principle, might drive convection up to the depth where the water column reaches 3.98 ${\;}^{\circ }$C. Systems exhibiting such thermal structures include Lakes BLH-A, Ngoring, and Ulansu (17, 23, 24, 66). This scenario is consistent with the WLC regime illustrated in Fig. 1A. However, direct evidence of convection is still lacking due to the absence of velocity measurements.

As winter progresses and snow cover diminishes, the cold, arid Tibetan air maintains surface ice while substantial solar radiation penetrates beneath it. Under these conditions, near-ice waters can warm well above 3.98 ${\;}^{\circ }$C, yielding temperature profiles characteristic of the TWLC regime (Fig. 1A). This top warm layer—even reaching 10 ${\;}^{\circ }$C in some cases—enhances the water–ice flux ${\bar{\Phi }}_{i}$ and accelerates ice melting, as reported in Lake Ulansu (24). To date, only one study has estimated—from indirect observations—the efficiency of solar radiation in controlling ${\bar{\Phi }}_{i}$, suggesting that up to 82% of its absorbed energy is transferred to the ice (24). Whether such high efficiency stems from active convection near the ice–water interface remains an unanswered question to which we can shed some light. Using the reported scaling laws for ${\bar{\Phi }}_{i}$ (Figs. 2 and 3) and the heat balance of our system (Supplementary material), we estimate this efficiency in our parameter space, obtaining a range of regime-dependent values (Fig. S12). The WLC and conductive regimes yield values exceeding 100%, as additional heat is injected into the water column through the sediments. The other convective regimes have values lower than 100%. However, we observe values strikingly close to 100% within the TWLC regime, which results from the confinement of its “warm” convective layer in the vicinity of the ice cover. Values close to 100% are also observed in parts of the CLC and DLC regimes, but they drop as $\varphi_{b}$ decreases.

### The role of salinity

In the absence of direct velocity measurements, salinity stratification has been hypothesized to explain temperature profiles resembling the DLC or TWLC regimes (19, 24, 66). Our results show that salinity is not required; such profiles can arise from the interplay of freshwater’s nonmonotonic EOS, solar heating, and sediment temperature. The stabilizing effectiveness of salinity gradients remains uncertain and is the subject of ongoing debate (19, 66).

Observations from lakes show that salinity gradients often intensify near the sediment–water interface, creating a locally stable stratification that may suppress convection in the deepest waters. Combining field data with numerical simulations, Mironov et al. (21) examined how dissolved salts modulate the thermal structure of radiatively heated ice-covered lakes. Their simulations—aimed primarily at reproducing evolving temperature profiles—suggest that salinity can stabilize a warm near-bottom layer with temperatures exceeding 3.98 ${\;}^{\circ }$C while allowing convection to persist in the overlying water column. Their measurements further show that a warm layer can also developed near the ice, again reaching temperatures above 3.98 ${\;}^{\circ }$C. However, limitations of their modeling framework precluded a definitive assessment of whether the observed coupled temperature–salinity distribution should be convectively active. We hypothesize that such conditions may give rise to the DLC regime reported here.

To further clarify the role of dissolved salt in thermally forced under-ice waters, we ask how the transient dynamic in Fig. 4 changes when the water column contains a background salinity field that increases from the ice toward the sediment. To address this question, we extend our model by adding an advection–diffusion equation for salinity, fully coupled to the momentum transport through the EOS (Fig. S3). For direct comparison with our freshwater reference case (Fig. 4), we consider $\varphi_{b}=0.8$ and Rc=46.8, and prescribe an idealized initial salinity profile that increases exponentially from S=0 ppt at the ice–water boundary to S=2‰ at the bottom, consistent with observations in Tibetan lakes (eg 24). To probe conditions with warmer bottoms ($\varphi_{b}>1$), we perform an additional transient simulation with $\varphi_{b}=1.4$ and Rc=22.45 (details in Supplementary material). The simulations support the field-based expectation that salt-enriched deep waters are stabilized, producing a persistent pycnocline that limits convective exchange with the benthic layer. Importantly, above the pycnocline—where the stratification weakens and radiative heating can still generate unstable buoyancy—convection develops in close analogy to the freshwater scenarios. Across the transient evolution we again identify the CLC, DLC, TWLC, and WLC regimes (Figs. S15 and S16), demonstrating that salinity reorganizes the stratification without eliminating the fundamental convective phenomenology reported here. These results therefore provide a proof of concept that salt acts primarily as a modifier; it shifts the depth range, intensity, and parameter boundaries of the convective regimes, rather than precluding their emergence.

### Melting rates

As mentioned earlier, the water–ice heat flux warms the ice-cover, yet its contribution to the ice evolution remains under active investigation (24). Using a simplified version of the Stefan problem (Materials and methods)—which models the phase change of water owing to heat exchange—we can estimate melting rates directly from the water–ice heat flux data presented in Fig. 2.

For example, fixing $\varphi_{b}=0.8$ and considering cases with Rc≈28.1 and Rc≈74.8 (equivalent to 47.8 and 144.9 $W\;m^{-2}$), we obtain melting rates of 13.5 and 40.9 $\mathrm{mm}\;\mathrm{da}y^{-1}$, respectively. The former falls within the CLC regime, while the latter corresponds to the DLC regime—helping to explain the enhanced melting in the higher Rc case. Additionally, for a higher bottom temperature of $\varphi_{b}=1.4$ and Rc=2.8, the resulting upward heat flux is 79 $W\;m^{-2}$, yielding a melting rate of 22.4 $\mathrm{mm}\;\mathrm{da}y^{-1}$. These values are consistent with conditions observed in Lake Ulansu, one of the Tibetan heat collectors (24). Together, these examples highlight the predictive power of the scaling laws developed in this study, which enable quantitative estimates of melting rates across diverse thermal regimes using under-ice solar absorption and bottom temperature as inputs.

This study reveals and unifies a spectrum of dynamic regimes—some previously observed, others theoretically predicted but not yet confirmed—within a canonical framework that connects theoretical insights, numerical simulations, and in situ observations in ice-covered lakes.

## Materials and methods

### Mathematical formulation

The conceptual model is governed by the conservation of mass, momentum, and heat, for a Newtonian, incompressible fluid. Considering the temperature and water depth rage of interest, we base our framework in the widely validated Oberbeck–Boussinesq approximation. Based on the same arguments, we adopt a quadratic EOS (Fig. S1). The system is forced by the absorption of solar radiation—following a Beer–Lambert model—included as a source term in the heat equation. Our dimensional analysis considers length L, time T, and velocity V scales


(7) $$L=h,\;T=\sqrt{h/g\left(1-\rho_{i}/\rho_{r}\right)},\;V=L/T,$$

where *g* is the gravitational acceleration, while $\rho_{i}$ and $\rho_{r}$ are, respectively, the liquid water densities at the freezing point and the temperature of maximum density. The dimensionless equations governing the evolution of the velocity u, temperature φ, and modified pressure $p_{*}$, in time *t* and space $x=x\hat{x}+y\hat{y}+z\hat{z}$, are


(8a ) ∇∙u=0,

(8b ) $$\frac{\partial u}{\partial t}+\left(\nabla u\right)u=-\nabla p_{*}-{\left(\varphi -1\right)}^{2}\hat{z}+\frac{1}{\sqrt{\mathrm{Gr}}}\nabla^{2}u,$$

(8c ) $$\frac{\partial \varphi }{\partial t}+\nabla \varphi ∙u=\frac{1}{\mathrm{Pr}\sqrt{\mathrm{Gr}}}\nabla^{2}\varphi +\frac{\mathrm{Rc}}{\Lambda \mathrm{Pr}\sqrt{\mathrm{Gr}}}\exp\;\left(-\frac{z}{\Lambda }\right),$$

(8d ) $$u(z=0)=u(z=1)=0,\;\varphi (z=0)=0,\;\varphi (z=1)=\varphi_{b}.$$

The Prandtl number is defined as $\mathrm{Pr}=\mu c_{v}/k\approx 11.67$, where *μ* is the dynamic viscosity, *k* is the thermal conductivity, and $c_{v}$ is the specific isochoric heat capacity. The Grashof number is defined as $\mathrm{Gr}=\rho_{r}^{2}h^{3}g(1-\rho_{i}/\rho_{r})/\mu^{2}$.

### Parameter space

As mentioned in the main text, we fix Λ=0.1. We also fix $\mathrm{Gr}\approx 5.25\times 10^{8}$, consistent with a depth h=1m at the Earth’s standard gravity, enabling us to capture a wide range of dynamics regimes. Then, we concentrate our efforts on the parameters that control solar heating and sediment–water heat exchange. We vary $\varphi_{b}$ between 0 and 1.4, corresponding to bottom temperatures from 0 ${\;}^{\circ }$C to 5.57 ${\;}^{\circ }$C (24, 87). For Rc, values between 0.9 and 187.1 are considered, corresponding to downward solar irradiances from 2.1 to 418 $W\;m^{-2}$, covering and expanding the range reported in field studies (28). For completeness, the effect of the Grashof number on the convective regimes is further discussed in Supplementary material, supported by direct numerical simulations.

### Numerical experiments and computational resources

Direct numerical simulations are performed in 3D for a sound characterization of flow structures (Fig. 1), while 2D DNS are used for a comprehensive exploration of the parameter space (Fig. 2) and to illustrate long-term transient dynamics (Fig. 4). In all cases, the Dedalus spectral solver (88) is used to solve the governing equations (Eq. 8). A Chebyshev basis is used in the vertical axis, while a Fourier representation is used in the horizontal. In the 2D simulations, an 8:1 horizontal to vertical aspect ratio is used to ensure a robust characterization of large-scale structure and estimation of global quantities. A (2048×256) mesh is used to ensure well-resolved physics in terms of the Kolmogorov and Batchelor length scales (Fig. S8), while having consistently more than 10 grid points in the BLs (Fig. S7). The simulations are run until quasisteady states are reached (Fig. S5). For 3D simulations, the mesh size is 384×384×256, and different aspect ratios are used for each case: 3:1 for the DLC and WLC regimes, 4:1 for the WLC regime, and 1.7:1 for the TWLC regime. Time integration of the equations is performed by a third-order diagonally implicit Runge–Kutta scheme in the 2D simulations (89), while a second-order semi-implicit backward differentiation scheme is used for the 3D simulations (90).

The hardware used for the simulation consists of four computational nodes, each one equipped with two AMD EPYC 7713 CPUs, resulting in 128 physical cores per node. Each node has 500 GB of RAM, allowing us to use a total of 2TB for each 3D simulation.

### Spatial and time averaging

Spatial averages are performed within the Dedalus framework—while the simulations are running—to leverage the precision given by the spectral representation of the fields when performing differential and integral operations. On the other hand, the Simpson’s rule is used for the time integration of global quantities.

### Estimation of power laws coefficients

Using the data from the 2D simulations, the coefficients of the power laws for each regime were obtained by slightly different fitting methods. For the WLC and TLWC regimes (Eqs. 5 and 6), rearrangement and the use of logarithms allow fitting by least squares. In contrast, this method is not possible for the CLC and DLC regimes (Eqs. 3 and 4), requiring the use of nonlinear least squares.

### Estimation of melting rates

We consider a layer of isothermal ice of thickness $H^{\mathrm{ice}}$ and quantify the melting rate as $\left|dH^{\mathrm{ice}}/d\tilde{t}\right|$, where $\tilde{t}$ is the dimensional time. We compute the melting rate assuming that the water–ice heat flux (in dimensional form) $(\theta_{r}-\theta_{i}){\bar{\Phi }}_{i}/h$ obtained by our framework is the dominant component in the balance between latent heat and conduction at the ice–water interface. Then, from the Stefan condition, we obtain


(9) $$\left|\frac{dH^{\mathrm{ice}}}{d\tilde{t}}\right|=\frac{k\left(\theta_{r}-\theta_{i}\right)}{\rho^{\mathrm{ice}}\;\ell \;h}|{\bar{\Phi }}_{i}|,$$

where ℓ is the specific latent heat and $\rho^{\mathrm{ice}}$ is the density of ice at the freezing temperature.

## Supplementary Material

pgag045_Supplementary_Data

## Data Availability

All figures data are available at 10.5281/zenodo.17055415 (92). The Supplementary material includes a dataset with all parameters needed to reproduce our simulations—nondimensional numbers, grid resolution (number of grid points), and characteristic integration time scales. We ran the simulations with Dedalus, an open-source, Python-based, MPI-parallel spectral solver (88), available at https://github.com/DedalusProject/dedalus.
