# Supplementary material for: Under-ice convective regimes driven by sunlight and sediment temperature control water–ice heat flux
Source: PNAS Nexus. 2026 Mar 4;5(3):pgag045. doi: 10.1093/pnasnexus/pgag045 (PMC13016998; doi:10.1093/pnasnexus/pgag045)
Supplement: pgag045_Supplementary_Data [file pgag045_supplementary_data.zip › PNASNEXUS-PNASNEXUS-2025-01343-TR-s07.pdf]

Supporting Information for

Under-ice convective regimes driven by sunlight and sediment temperature control water–ice heat flux

Gustavo Estay, Daisuke Noto and Hugo N. Ulloa

Corresponding Author: Hugo N. Ulloa.
E-mail: ulloa@sas.upenn.edu

This PDF file includes:

Figs. S1 to S16
Table S1
Legends for Movies S1 to S5
Legends for Dataset S1 to S2
SI References

Other supporting materials for this manuscript include the following:

Movies S1 to S5
Datasets S1 to S2

Theoretical framework

Equation of state. For shallow freshwater at atmospheric pressure, the density ρ depends mainly on the temperature θ. Moreover, between the freezing point temperature θ_i and 8 °C the density of water can be expressed as

$$\rho(\theta) = \rho_\mathrm{r} - (\rho_\mathrm{r} - \rho_\mathrm{i}) \frac{(\theta - \theta_\mathrm{r})^2}{(\theta_\mathrm{r} - \theta_\mathrm{i})^2}, \qquad [1]$$

where ρ_r is the maximum density, ρ_i is the density of liquid water at the freezing point, and θ_r is the temperature of maximum density. At 101 325 Pa, the numerical values of the aforementioned parameters are (1)

$$\theta_\mathrm{i} \approx 0\,^\circ\mathrm{C}, \qquad \rho_\mathrm{i} \approx 999.843\,\mathrm{kg\,m^{-3}}, \qquad \theta_\mathrm{r} \approx 3.978\,^\circ\mathrm{C}, \qquad \rho_\mathrm{r} \approx 999.975\,\mathrm{kg\,m^{-3}}, \qquad [2]$$

which where obtained from the iapws Python implementation (2). Then, the equation of state considered in this work, Eq. (1), is shown in Fig. S1.

Governing equations. We consider a position vector $\tilde{\mathbf{x}} = \tilde{x}\hat{\mathbf{x}} + \tilde{y}\hat{\mathbf{y}} + \tilde{z}\hat{\mathbf{z}}$. The domain consists of an infinite layer of fluid bounded by the planes $\tilde{z} = 0$ and $\tilde{z} = h > 0$. These planes represent the ice-water interface and the bottom boundary, respectively. The frame of reference is defined in such a way that the acceleration of gravity can be written as $\mathbf{g} = g\hat{\mathbf{z}}$ ($g = 9.806\,65\,\mathrm{m\,s^{-2}}$).

Water is considered an incompressible fluid. Therefore, its velocity $\tilde{\mathbf{u}}$ satisfies the continuity equation

$$\nabla \bullet \tilde{\mathbf{u}} = 0. \qquad [3]$$

For the momentum equation, we consider the Oberbeck-Boussinesq approximation (3, 4), that is, we neglect variations of density in the inertia:

$$\rho_\mathrm{r} \left[\frac{\partial \tilde{\mathbf{u}}}{\partial \tilde{t}} + (\nabla \tilde{\mathbf{u}})\,\tilde{\mathbf{u}}\right] = -\nabla \tilde{p} + \rho g \hat{\mathbf{z}} + \mu \nabla^2 \tilde{\mathbf{u}}, \qquad [4]$$

where $\tilde{t}$, $\tilde{p}$ are time and pressure respectively, while μ is the dynamic viscosity at θ_r.

Combining the non-monotonic equation of state (Eq. 1) with the momentum balance (Eq. 4) we obtain

$$\frac{\partial \tilde{\mathbf{u}}}{\partial \tilde{t}} + (\nabla \tilde{\mathbf{u}})\,\tilde{\mathbf{u}} = -\frac{1}{\rho_\mathrm{r}} \nabla \tilde{p} + g\hat{\mathbf{z}} - g\left(1 - \frac{\rho_\mathrm{i}}{\rho_\mathrm{r}}\right) \frac{(\theta - \theta_\mathrm{r})^2}{(\theta_\mathrm{r} - \theta_\mathrm{i})^2} \hat{\mathbf{z}} + \frac{\mu}{\rho_\mathrm{r}} \nabla^2 \tilde{\mathbf{u}}. \qquad [5]$$

To simplify Eq. 5, we define the modified pressure $\tilde{p}_* = \tilde{p}/\rho_\mathrm{r} - g\tilde{z}$ to obtain

$$\frac{\partial \tilde{\mathbf{u}}}{\partial \tilde{t}} + (\nabla \tilde{\mathbf{u}})\,\tilde{\mathbf{u}} = -\nabla \tilde{p}_* - g\left(1 - \frac{\rho_\mathrm{i}}{\rho_\mathrm{r}}\right) \frac{(\theta - \theta_\mathrm{r})^2}{(\theta_\mathrm{r} - \theta_\mathrm{i})^2} \hat{\mathbf{z}} + \frac{\mu}{\rho_\mathrm{r}} \nabla^2 \tilde{\mathbf{u}}. \qquad [6]$$

No-slip and no-penetration boundary conditions for the velocity are considered:

$$\tilde{\mathbf{u}}(\tilde{z} = 0) = \tilde{\mathbf{u}}(\tilde{z} = h) = \mathbf{0}. \qquad [7]$$

For the heat equation, we maintain the main assumptions of the Oberbeck–Boussinesq approximation, that is, neglecting density variations and viscous dissipation. Besides heat conduction, we also consider a body heat source in the balance:

$$\rho_\mathrm{r} c_\mathrm{v} \left[\frac{\partial \theta}{\partial \tilde{t}} + \nabla \theta \bullet \mathbf{u}\right] = k \nabla^2 \theta + \rho_\mathrm{r} r. \qquad [8]$$

The coefficients c_v and k are, respectively, the specific isochoric heat capacity and the thermal conductivity, both evaluated at $\theta = \theta_\mathrm{r}$. The variable r is the specific rate of body heat transfer that, in our case, represents energy absorption from solar radiation. We consider a downwelling irradiance E with a value $E_0 = E(\tilde{z} = 0)$ at the ice-water interface. Then, in analogy with the Beer–Lambert law, we have the following model (5)

$$\rho_\mathrm{r} r = -\frac{\partial E}{\partial \tilde{z}} = K_\mathrm{d} E \Longrightarrow \rho_\mathrm{r} r = K_\mathrm{d} E_0 \exp(-K_\mathrm{d} \tilde{z}), \qquad [9]$$

where K_d is the diffuse attenuation coefficient. Strictly speaking, K_d is wavelength-dependent (6, 7), however, for simplicity, K_d represents an integrated value across all light wavelengths.

From Eq. 8 and Eq. 9, the heat equation can be written as

$$\frac{\partial \theta}{\partial \tilde{t}} + \nabla \theta \bullet \mathbf{u} = \frac{k}{\rho_\mathrm{r} c_\mathrm{v}} \nabla^2 \theta + \frac{K_\mathrm{d} E_0}{\rho_\mathrm{r} c_\mathrm{v}} \exp(-K_\mathrm{d} \tilde{z}). \qquad [10]$$

We consider the following boundary conditions for the temperature field,

$$\theta(\tilde{z} = 0) = \theta_\mathrm{i}, \qquad \theta(\tilde{z} = h) = \theta_\mathrm{b}, \qquad [11]$$

where θ_i is the freezing point temperature and $\theta_\mathrm{b} \geq \theta_\mathrm{i}$ is a fixed temperature at the bottom of the water body.

Dimensional analysis. We start by defining the following dimensionless variables:

$$t=\tilde{t}\bigg/\sqrt{\frac{h}{g\left(1-\rho_{\mathrm{i}}/\rho_{\mathrm{r}}\right)}},\qquad \mathbf{x}=\frac{\tilde{\mathbf{x}}}{h},\qquad \mathbf{u}=\frac{\tilde{\mathbf{u}}}{\sqrt{g\left(1-\rho_{\mathrm{i}}/\rho_{\mathrm{r}}\right)h}},\qquad p_*=\frac{\tilde{p}_*}{g\left(1-\rho_{\mathrm{i}}/\rho_{\mathrm{r}}\right)h},\qquad \varphi=\frac{\theta-\theta_{\mathrm{i}}}{\theta_{\mathrm{r}}-\theta_{\mathrm{i}}}. \tag{12}$$

The equation of state can be written as

$$\rho=\rho_{\mathrm{r}}-\left(\rho_{\mathrm{r}}-\rho_{\mathrm{i}}\right)\left(\varphi-1\right)^2, \tag{13}$$

then, the dimensionless governing equations are

$$\nabla\bullet\mathbf{u}=0, \tag{14a}$$

$$\frac{\partial\mathbf{u}}{\partial t}+\left(\nabla\mathbf{u}\right)\mathbf{u}=-\nabla p_*-\left(\varphi-1\right)^2\hat{\mathbf{z}}+\frac{1}{\sqrt{\mathrm{Gr}}}\nabla^2\mathbf{u}, \tag{14b}$$

$$\frac{\partial\varphi}{\partial t}+\nabla\varphi\bullet\mathbf{u}=\frac{1}{\mathrm{Pr}\sqrt{\mathrm{Gr}}}\nabla^2\varphi+\frac{\mathrm{Rc}}{\Lambda\mathrm{Pr}\sqrt{\mathrm{Gr}}}\exp\left(-\frac{z}{\Lambda}\right), \tag{14c}$$

with boundary conditions

$$\mathbf{u}\left(z=0\right)=\mathbf{0},\qquad \mathbf{u}\left(z=1\right)=\mathbf{0},\qquad \varphi\left(z=0\right)=0,\qquad \varphi\left(z=1\right)=\varphi_{\mathrm{b}}, \tag{15}$$

and the dimensionless parameters that describe the system are

$$\mathrm{Pr}=\frac{\mu c_{\mathrm{v}}}{k},\qquad \mathrm{Rc}=\frac{E_0 h}{k\left(\theta_{\mathrm{r}}-\theta_{\mathrm{i}}\right)},\qquad \mathrm{Gr}=\frac{\rho_{\mathrm{r}}^2 h^3 g\left(1-\rho_{\mathrm{i}}/\rho_{\mathrm{r}}\right)}{\mu^2},\qquad \Lambda=\frac{1}{hK_{\mathrm{d}}},\qquad \varphi_{\mathrm{b}}=\frac{\theta_{\mathrm{b}}-\theta_{\mathrm{i}}}{\theta_{\mathrm{r}}-\theta_{\mathrm{i}}}. \tag{16}$$

Since this model is specific for water, the Prandtl number Pr is fixed, while the radiative-conductive number Rc, the Grashof number Gr, the dimensionless e-folding depth Λ and the dimensionless bottom temperature φ_{b} are the controlling parameters of the system.

Averages. Let's consider a field $\psi(\mathbf{x},t)$. The following notation will be used for different averages of ψ in our domain:

$$\langle\psi\rangle=\frac{\iiint\varphi\,\mathrm{d}x\,\mathrm{d}y\,\mathrm{d}z}{\iiint 1\,\mathrm{d}x\,\mathrm{d}y\,\mathrm{d}z},\qquad \langle\psi\rangle_{xy}=\frac{\iint\varphi\,\mathrm{d}x\,\mathrm{d}y}{\iint 1\,\mathrm{d}x\,\mathrm{d}y}, \tag{17}$$

For time average we have

$$\bar{\psi}=\frac{1}{\tau}\int_t^{t+\tau}\psi(\mathbf{x},s)\,\mathrm{d}s, \tag{18}$$

where τ represents an integration time window long enough to average 100 convetive timescale $\tau_c=\sqrt{h/(g(1-\rho_i/\rho_r))}$

Heat budget. Let's define the following global quantities:

$$\Phi_{\mathrm{i}}=\frac{-\iint\partial_z\varphi|_{z=0}\,\mathrm{d}x\,\mathrm{d}y}{\iiint\mathrm{d}x\,\mathrm{d}y\,\mathrm{d}z},\qquad \Phi_{\mathrm{b}}=\frac{\iint\partial_z\varphi|_{z=1}\,\mathrm{d}x\,\mathrm{d}y}{\iiint\mathrm{d}x\,\mathrm{d}y\,\mathrm{d}z}. \tag{19}$$

Integrating the dimensionless heat equation (Eq. 14c) in the domain, we obtain the transient heat budget

$$\frac{\mathrm{d}\langle\varphi\rangle}{\mathrm{dt}}=\frac{1}{\mathrm{Pr}\sqrt{\mathrm{Gr}}}\left(\Phi_{\mathrm{i}}+\Phi_{\mathrm{b}}\right)+\frac{\mathrm{Rc}}{\mathrm{Pr}\sqrt{\mathrm{Gr}}}\left[1-\exp\left(-1/\Lambda\right)\right] \tag{20}$$

Integrating in time and assuming quasi-steady state conditions, we obtain

$$0=\frac{1}{\mathrm{Pr}\sqrt{\mathrm{Gr}}}\left(\bar{\Phi}_{\mathrm{i}}+\bar{\Phi}_{\mathrm{b}}\right)+\frac{\mathrm{Rc}}{\mathrm{Pr}\sqrt{\mathrm{Gr}}}\left[1-\exp\left(-1/\Lambda\right)\right]. \tag{21}$$

Conductive state. Let's consider the following assumptions: steady-state fields, zero velocity, temperature field independent of x and y. Then the dimensionless heat equation (Eq. 14c) is reduced to an ordinary differential equation

$$0=\frac{\mathrm{d}^2\varphi^{\mathrm{cond}}}{\mathrm{d}z^2}+\frac{\mathrm{Rc}}{\Lambda}\exp\left(-z/\Lambda\right), \tag{22}$$

that can be integrated to obtain a steady-state conductive temperature profile φ^{cond}:

$$\varphi^{\mathrm{cond}}=-\mathrm{Rc}\Lambda\exp\left(-z/\Lambda\right)+\left(\varphi_{\mathrm{b}}-\mathrm{Rc}\Lambda\left[1-\exp\left(-1/\Lambda\right)\right]\right)z+\mathrm{Rc}\Lambda, \tag{23}$$

$$\frac{\mathrm{d}\varphi^{\mathrm{cond}}}{\mathrm{d}z}=\mathrm{Rc}\exp\left(-z/\Lambda\right)+\varphi_{\mathrm{b}}-\mathrm{Rc}\Lambda\left[1-\exp\left(-1/\Lambda\right)\right]. \tag{24}$$

We also have expressions for the conductive boundary heat fluxes:

$$\Phi_{\mathrm{i}}^{\mathrm{cond}}=\mathrm{Rc}\Lambda\left[1-\exp\left(-1/\Lambda\right)\right]-\mathrm{Rc}-\varphi_{\mathrm{b}},\qquad \Phi_{\mathrm{b}}^{\mathrm{cond}}=\mathrm{Rc}\exp\left(-1/\Lambda\right)-\mathrm{Rc}\Lambda\left[1-\exp\left(-1/\Lambda\right)\right]+\varphi_{\mathrm{b}}. \tag{25}$$

Stability of the conductive state. The conductive state is stable when the following condition for the conductive density profile ρ^{cond} is satisfied through the water column $(0 \le z \le 1)$

$$\frac{\mathrm{d}\rho^{\mathrm{cond}}}{\mathrm{d}z} > 0 \Longleftrightarrow \left(1 - \varphi^{\mathrm{cond}}\right) \frac{\mathrm{d}\varphi^{\mathrm{cond}}}{\mathrm{d}z} > 0 \qquad [26]$$

From Eq. 22 is concluded that $-\varphi^{\mathrm{cond}}$ is a strongly convex function. As a result, φ^{cond} has a unique global maximum $\varphi_*^{\mathrm{cond}} = \varphi^{\mathrm{cond}}(z_*)$. Moreover, the sign of $\mathrm{d}\varphi^{\mathrm{cond}}/\mathrm{d}z$ is given by

$$\frac{\mathrm{d}\varphi^{\mathrm{cond}}}{\mathrm{d}z} \begin{cases} \ge 0 & z \le z_* \\ < 0 & z > z_* \end{cases} \qquad [27]$$

If $z_* \ge 1$, $\mathrm{d}\varphi^{\mathrm{cond}}/\mathrm{d}z$ does not change its sing in our domain. If $\varphi_b \le 1$ also holds, then the stability condition (Eq. 26) is satisfied.

From Eq. 24 we obtain that

$$z_* = -\Lambda \ln \left(\Lambda \left[1 - \exp\left(-1/\Lambda\right)\right] - \frac{\varphi_{\mathrm{b}}}{\mathrm{Rc}} \right). \qquad [28]$$

It is concluded that the following conditions occurring simultaneously guarantee the stability of the conductive profile

$$0 \le \varphi_{\mathrm{b}} \le 1, \qquad 0 \le \mathrm{Rc} \le \frac{\varphi_{\mathrm{b}}/\Lambda}{1 - \left(1 + 1/\Lambda\right)\exp\left(-1/\Lambda\right)}. \qquad [29]$$

In that case, it can be said that the conductive profile (Eq. 23) is unconditionally stable, since its stability is independent of the Grashof number of the system. Examples of conductive profiles are shown in Fig. S2; one stable and three unstable profiles not fulfilling the stability conditions (Eq. 29) in different ways.

Microscales. For incompressible fluids, the average viscous dissipation per unit of mass can be computed as

$$\langle\tilde{\varepsilon}\rangle = \frac{\mu}{\rho} \left\langle (\nabla\tilde{\mathbf{u}}) \bullet (\nabla\tilde{\mathbf{u}}) \right\rangle. \qquad [30]$$

Considering a time-integrated viscous dissipation, the global Kolmogorov and Batchelor length scales can be defined, respectively, as

$$\tilde{\eta}_{\mathrm{K}} = \left(\frac{\mu^3}{\rho^3 \overline{\langle\tilde{\varepsilon}\rangle}} \right)^{1/4}, \qquad \tilde{\eta}_{\mathrm{B}} = \frac{\tilde{\eta}_{\mathrm{K}}}{\sqrt{\mathrm{Pr}}}. \qquad [31]$$

In our context, we consider the corresponding dimensionless microscales

$$\eta_{\mathrm{K}} = \frac{\tilde{\eta}_{\mathrm{K}}}{h} = \left(\mathrm{Gr}\overline{\langle(\nabla\mathbf{u}) \bullet (\nabla\mathbf{u})\rangle} \right)^{-1/4}, \qquad \eta_{\mathrm{B}} = \frac{\tilde{\eta}_{\mathrm{B}}}{h} = \left(\mathrm{Pr}^2\mathrm{Gr}\overline{\langle(\nabla\mathbf{u}) \bullet (\nabla\mathbf{u})\rangle} \right)^{-1/4}. \qquad [32]$$

Extended mathematical model with dissolved salts. For low salinity values, the following model can be adopted for the density equation of state,

$$\rho\left(\theta, S\right) = \rho_{\mathrm{r}} - \left(\rho_{\mathrm{r}} - \rho_{\mathrm{i}}\right) \frac{\left(\theta - \theta_{\mathrm{r}}\right)^2}{\left(\theta_{\mathrm{r}} - \theta_{\mathrm{i}}\right)^2} + \alpha S, \qquad [33]$$

where S is the salinity and $\alpha \approx 800.11\,\mathrm{kg\,m^{-3}}$ is obtained fitting data (2) in the in range of conditions relevant for this study (Fig. S3).

Fixing the bottom salinity S_{b}, we can define the normalized salinity $s = S/S_{\mathrm{b}}$, and consider the nondimensional EOS

$$\frac{\rho}{\rho_{\mathrm{r}}} = 1 - \left(1 - \frac{\rho_{\mathrm{i}}}{\rho_{\mathrm{r}}}\right)\left(\varphi - 1\right)^2 + \frac{\alpha S_{\mathrm{b}}}{\rho_{\mathrm{r}}} s. \qquad [34]$$

which, in turn, results in a modified nondimensional momentum equation

$$\frac{\partial\mathbf{u}}{\partial t} + \left(\nabla\mathbf{u}\right)\mathbf{u} = -\nabla p_* - \left(\varphi - 1\right)^2\hat{\mathbf{z}} + \mathrm{A}s\hat{\mathbf{z}} + \frac{1}{\sqrt{\mathrm{Gr}}}\nabla^2\mathbf{u}, \qquad [35]$$

while the transport of normalized salinity is given by

$$\frac{\partial s}{\partial t} + \nabla s \bullet \mathbf{u} = \frac{1}{\mathrm{Sc}\sqrt{\mathrm{Gr}}}\nabla^2 s \qquad [36]$$

As a result, two additional dimensionless parameters are required for the model,

$$\mathrm{A} = \frac{\alpha S_{\mathrm{b}}}{\rho_{\mathrm{r}} - \rho_{\mathrm{i}}}, \qquad \mathrm{Sc} = \frac{\mu}{\rho_{\mathrm{r}} D}, \qquad [37]$$

where D is the mass diffusivity of salt and Sc is the Schmidt number. We adopt the value $\mathrm{Sc} \approx 2091.24$. The boundary conditions for salinity are $s\left(z = 0\right) = 0$ and $s\left(z = 1\right) = 1$.

Numerical experiments

A set of numerical experiments has been performed to understand the quasi-steady-state behavior of the system for different values of the controlling parameters. The dimensionless governing equations (Eq. 14) are solved directly using the Dedalus spectral solver (8). A Chebyshev basis is used in the vertical axis to allow us to impose Dirichlet boundary conditions (Eq. 15) and achieve well-resolved boundary layers, while a Fourier representation is used in the horizontal.

In this work, we focus on the effect of radiation and the bottom temperature on the system, so we keep the other parameters constant in our set of numerical simulations. The numerical values used are

$$\mathrm{Pr} \approx 11.67 \qquad \mathrm{Gr} \approx 525379389.14 \qquad \Lambda = 0.1 \tag{38}$$

We perform three-dimensional simulations to obtain a realistic representation of the flow structures and two-dimensional simulations to have a comprehensive coverage of the parameter space.

Three-dimensional numerical experiments. . Four three-dimensional numerical simulations were performed to illustrate the spatial and temporal variability of each of the four under-ice convective regimes at quasi-steady-state, as identified here. Snapshots of the temperature fluctuations for each of these regimes are provided in the main text, Figs. 1B, C, D, E. In addition, we provide supplementary Movies S1-S4. The parameters utilized for each simulation are reported in Table S1.

Two-dimensional numerical experiments. To obtain a robust representation of the quasi-steady state of the system in an efficient way, we perform the numerical simulations in two stages. First, we run a simulation in a coarse mesh (128×128) with an aspect ratio $4:1$ until $t = 10^5$. The resulting fields are interpolated into a finer mesh (2048×256) with aspect ratio $8:1$, and a simulation is run for $5 \cdot 10^4$ units of dimensionless time. This finer mesh ensures that the boundary layers as well as the microscales η_K and η_B in Eq. (31) are well resolved—as discussed below. Whereas the aspect ratio ensures a sound characterization and statistics of the largest convective structures. While the latter simulation is running, time series of relevant global quantities like $\langle \varphi \rangle$, Φ_i, Φ_b are computed, taking advantage of the spectral representation from the solver. Moreover, the time averages (Eq. 18) $\bar{\Phi}_\mathrm{i}$ and $\bar{\Phi}_\mathrm{b}$ are computed using a time window of $5 \cdot 10^3$ (Fig. S4).

Based on the quasi steady-state heat budget (Eq. 21) we define the following metric

$$\frac{-\bar{\Phi}_\mathrm{i} - \bar{\Phi}_\mathrm{b}}{\mathrm{Rc}\left[1 - \exp\left(-1/\Lambda\right)\right]}, \tag{39}$$

that represents the ratio between the heat conduction at the boundaries and the absorption of solar radiation. A value of 1 corresponds to a fully closed quasi-steady-state heat budget. It is found that in our two-dimensional simulations the heat budget is closed within a tolerance of around 1 % (Fig. S5).

We perform an *a posteriori* check of the number of grid points contained on the top and bottom thermal boundary layers. To compute the thickness of a boundary layer, we first consider the value φ_∞, defined as the closest point to the boundary in which the interpolated temperature profile has null derivative (9). Then, the slope of the temperature profile at the boundary is projected and intersected with φ_∞. The boundary layer is defined as the region between the boundary and the aforementioned intersection (Fig. S6). The number of points within the boundary layers for each simulation is shown in Fig. S7.

To check that the spatial resolution achieved by the finer mesh allows the turbulent flow scales to be well resolved, we consider the criterion by Grotzbach (10) for $\mathrm{Pr} \geq 1$, considered as a standard for direct numerical simulations (11–13). This criterion can be written in dimensionless form as

$$\Delta = \left(\Delta_x \Delta_z\right)^{1/2} \leq \pi \eta_\mathrm{B}, \tag{40}$$

where Δ_x is the dimensionless distance between two adjacent grid points along the x axis, and Δ_z is the dimensionless distance between two adjacent grid points along the z axis. Since this requirement needs to be fulfilled for any pair of grid points, we define Δ as the worst-case scenario, that is, the highest value in the mesh. Furthermore, thermal boundary layers, which are smaller than the viscous boundary layer, were resolved by more than 10 grid points (Fig. S6 and Fig. S7), following the standard of previous numerical studies on Rayleigh-Bénard Convection (14, 15)

A. The effect of the Grashof number. The Grashof number, $\mathrm{Gr} = h^3 g(1 - \rho_\mathrm{i}/\rho_\mathrm{r})/(\mu/\rho_\mathrm{r})^2$, quantifies the ratio of buoyancy driving to viscous resistance and is one of the five control parameters in our nondimensional formulation (Eq. 16). By construction, $\mathrm{Gr} \propto h^3$, where h is the water depth. Depth variations therefore map directly onto orders-of-magnitude changes in Gr: shallow systems correspond to smaller Gr, where viscous stresses exert a stronger relative constraint on motion, whereas deeper systems attain larger Gr and more readily sustain buoyancy-driven circulation. This dependence can reshape the convective dynamics—e.g.intensity and flow morphology—while leaving the purely conductive regime unchanged, consistent with our theoretical prediction (Eq. 23). Below, we expand on the physical and mathematical implications of varying Gr for the regime structure of the system.

From the steady-state heat balance of the system, it yields:

$$0 = \bar{\Phi}_\mathrm{i} + \bar{\Phi}_\mathrm{b} + \mathrm{Rc}\left[1 - \exp\left(-1/\Lambda\right)\right] \Longrightarrow \frac{\partial \bar{\Phi}_\mathrm{i}}{\partial \mathrm{Gr}} = -\frac{\partial \bar{\Phi}_\mathrm{b}}{\partial \mathrm{Gr}}.$$

This identity makes explicit that the net radiative heat input to the water column is fully prescribed by Rc and Λ and, in steady state, is independent of Gr. What the budget does not determine is how that fixed radiative input is partitioned between the ice–water heat flux, $\bar{\Phi}_{\mathrm{i}}$, and the bottom heat flux, $\bar{\Phi}_{\mathrm{b}}$. That partition is set by the dynamics and, in general, depends on Gr through its control of buoyancy-driven motion and viscous dissipation. Physically, increasing Gr strengthens convective transport relative to viscous resistance, thereby enhancing thermal mixing and redistributing heat between the upper and lower boundaries in a regime-dependent manner.

To complement our analysis and quantify how Gr controls the redistribution of heat between the ice–water and bottom boundaries, we carried out four additional direct numerical simulations—one representative case for each convective regime. In these runs, we decrease the Grashof number by one order of magnitude relative to the baseline value used throughout the study, thereby increasing the relative influence of viscous resistance and weakening buoyancy-driven transport. This controlled perturbation provides a clean sensitivity test: by comparing the resulting temperature profiles and boundary heat fluxes against the reference cases, we directly isolate the role of Gr in setting the thermal structure and the partitioning of heat export between the top and bottom boundaries. Results are reported in Fig. S13 and Fig. S14.

In the CLC, WLC, and TWLC regimes, the mean temperature of the convective mixing layer decreases as Gr increases (Fig. S13C, B, D). The physical mechanism is straightforward: a larger Gr strengthens advective transport relative to viscous damping, which promotes more efficient vertical homogenization and reduces the degree of thermal "storage" within the mixed layer, shifting heat export to the boundaries. The DLC regime is intrinsically richer because the flow organizes into two dynamically coupled convective layers separated by a stably stratified interface. In this case, increasing Gr enhances interlayer exchange and weakens the thermal contrast between the upper and lower mixed layers. Consequently, the upper convective layer cools (its mean temperature decreases), whereas the lower convective layer warms (its mean temperature increases), as the two layers are driven toward a more uniform thermal state (Fig. S13A).

The analysis above indicates that Gr shifts specific regime boundaries by modulating the efficiency of convective mixing and, therefore, the radiative forcing required to reach key thermodynamic thresholds. In particular, both the CLC–DLC and WLC–TWLC transitions move to larger values of Rc as Gr increases: stronger mixing redistributes heat more effectively and delays (i) the emergence of temperatures exceeding the temperature of maximum density in the interior (CLC–DLC), and (ii) the point at which the radiatively driven warming overcomes the influence of the imposed bottom condition (WLC–TWLC). By contrast, the DLC–TWLC transition is essentially insensitive to Gr because it is set by the thermodynamic criterion $\varphi_{\mathrm{b}} = 1$.

In the WLC regime, the water column is energized not only by radiative absorption but also by conductive heat input from the bottom boundary. Increasing Gr intensifies convective transport, thins the thermal boundary layers, and thereby enhances interfacial heat transfer. The net effect is a larger upward heat flux from the bottom, which—at steady state—must be balanced by an increased heat loss through the ice–water interface. In the other convective regimes, however, both boundaries can act as sinks (i.e., net cooling at the top and bottom), so the response of the heat-flux partition to variations in Gr is inherently more system- and regime-dependent and cannot be inferred from the global heat balance alone.

The effect of salinity. We performed two transient direct numerical simulations based on the extended mathematical model with dissolved salts (Eqs. 34-36). To reproduce the strong bottom saline stratification observed in some natural systems, we consider the following initial condition

$$s\,(t=0,z) = \exp\left[-10\,(1-z)\right]. \tag{41}$$

We consider a value $\mathrm{A} \approx 12.14$ (Eq. 37), consistent with a bottom salinity $S_{\mathrm{b}} = 2\times10^{-3}$ (2 ‰) (16). The first case considers a cold bottom temperature, $\varphi_{\mathrm{b}} = 0.8$, while the second considers a warm one, $\varphi_{\mathrm{b}} = 1.4$. The results are summarized in Fig. S15 and Fig. S16.

In both cases, the density stratification produced by the salinity distribution is able to stabilize a layer of fluid adjacent to the sediments. Nonetheless, convection develops above this layer, with dynamics closely analogous to those obtained for freshwater scenarios. Therefore, the most notorious effect of salinity is a reduction of convective region thickness. Another important consequence is that the sediment temperature φ_{b} can no longer be treated as a direct control parameter for the transition between convective regimes. In this context, the relevant parameter is the temperature at the top of the saline-stratified layer. Because this effective bottom temperature evolves in time as the salinity and temperature field adjust, it introduces an additional source of unsteadiness, making the analysis of transient dynamics more complex.

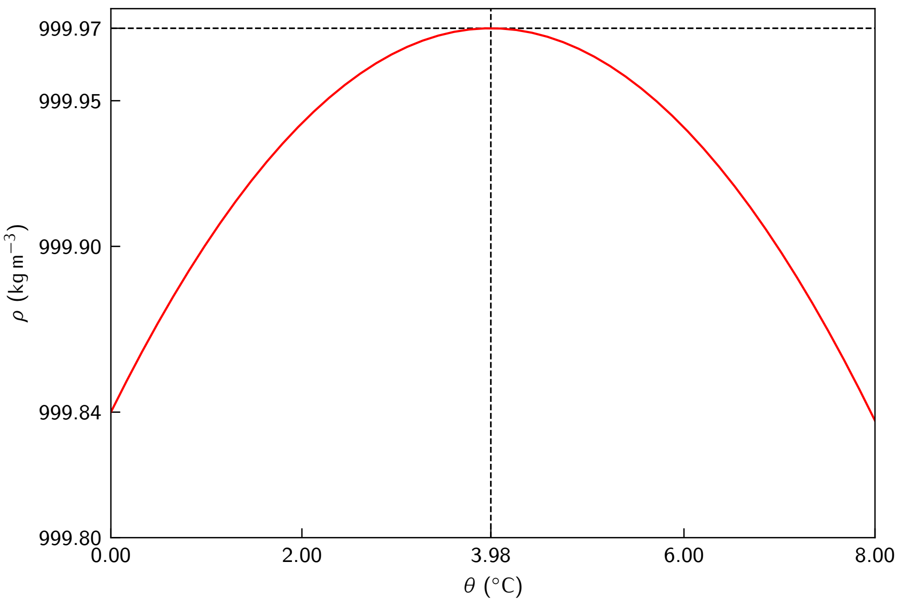

Fig. S1. Equation of state (EOS): relation between water density and temperature (Eq. 1) at atmospheric pressure $(101\,325\,\mathrm{Pa})$, between $0\,^{\circ}\mathrm{C}$ and $8\,^{\circ}\mathrm{C}$. Maximum density is achieved at $\theta_{\mathrm{r}} \approx 3.98\,^{\circ}\mathrm{C}$.

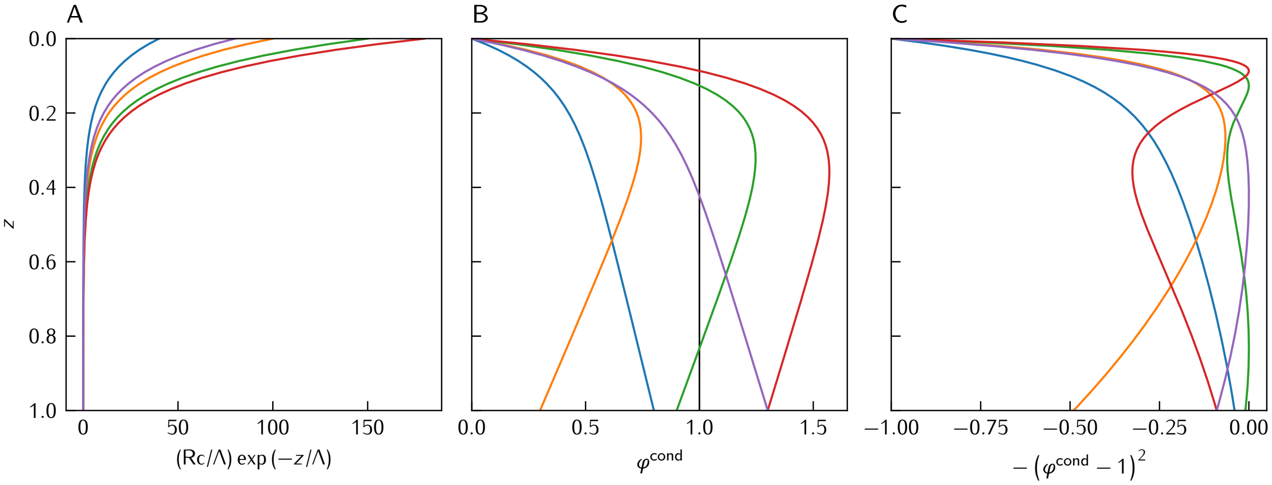

Fig. S2. Examples of (A) vertical distribution of solar energy absorption relevant for the conductive regime (Eq. 22), (B) their corresponding dimensionless temperature profiles (Eq. 23), and (C) their dimensionless buoyancy term (Eq. 14b) with respect to the reference density. A stable conductive profile is shown in blue, while the others exhibit unstable density distributions (Eq. 29).

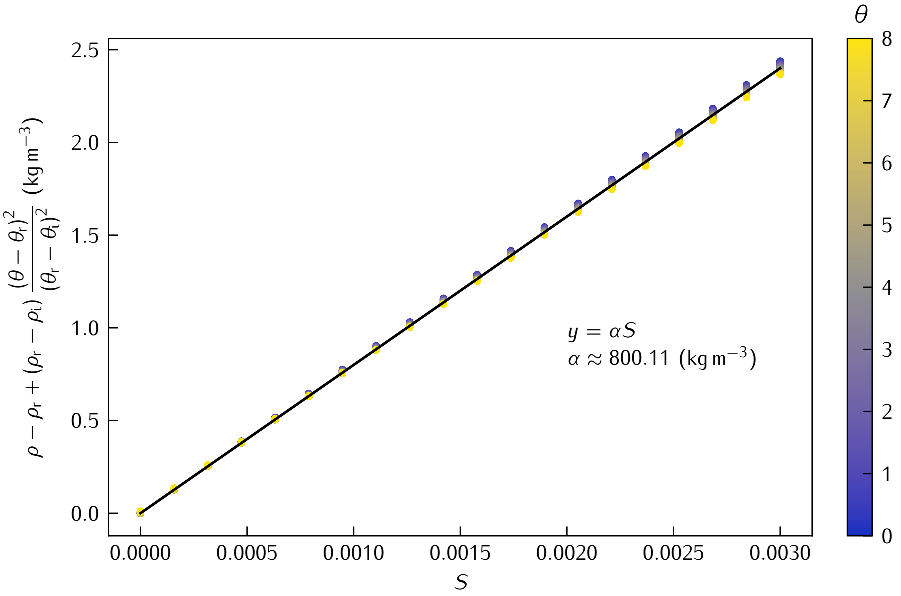

Fig. S3. Effect of salinity on the EOS and fitting of the coefficient α, at atmospheric pressure $(101\,325\,\mathrm{Pa})$, temperature range between $0\,^\circ\mathrm{C}$ and $8\,^\circ\mathrm{C}$, and salinity range between 0 and 0.003.

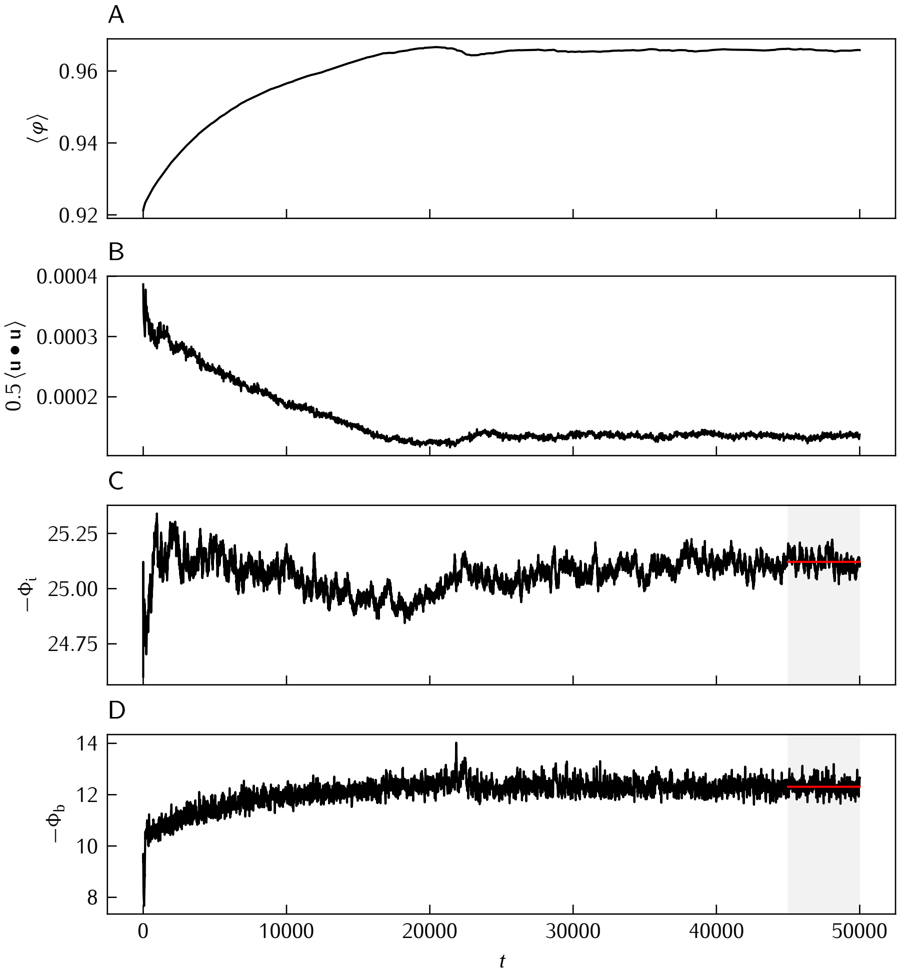

Fig. S4. Time evolution of dimensionless global quantities for a two-dimensional simulation ($\mathrm{Rc} \approx 37.41$, $\varphi_\mathrm{b} = 0.8$). The time averages $-\bar{\Phi}_\mathrm{i}$ and $-\bar{\Phi}_\mathrm{b}$ are shown in red, with gray shading indicating the integration window.

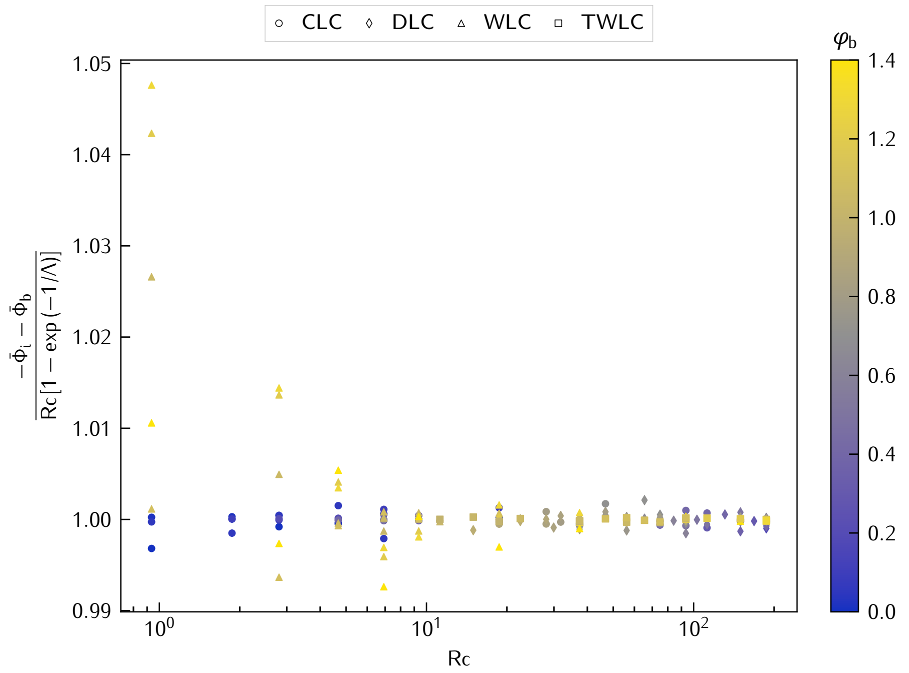

Fig. S5. Quasi steady-state heat budget closure (Eq. 39) for the two dimensional simulations. The color scale shows the value of the bottom temperature φ_b, while Rc is shown on the horizontal axis. Markers indicate the convective regime for each case.

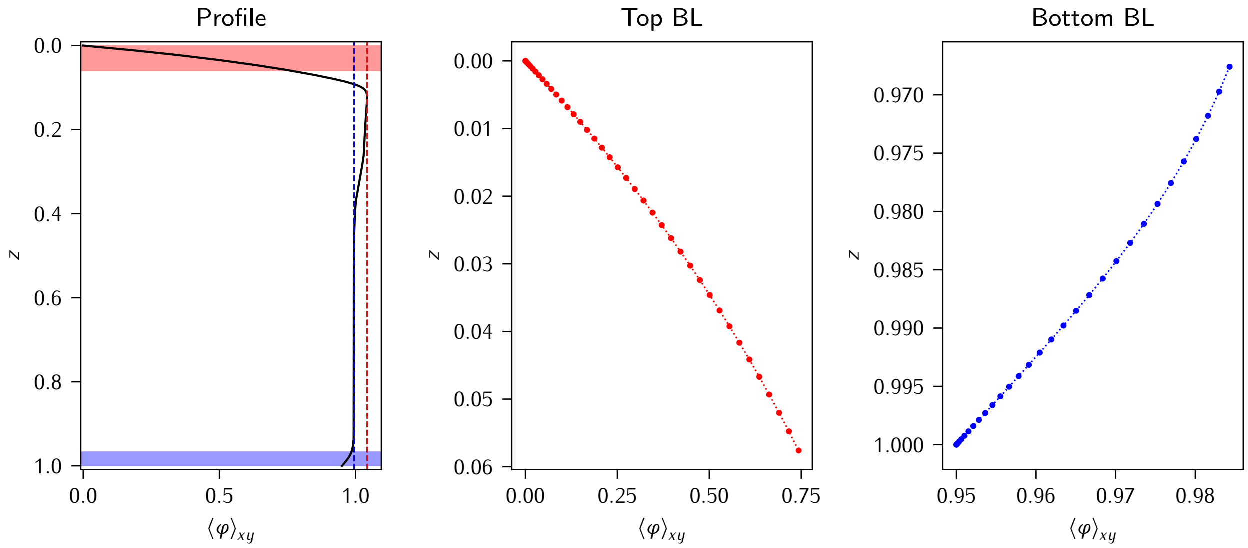

Fig. S6. Top and bottom boundary layers (BL) for a two-dimensional simulation ($\mathrm{Rc} \approx 18.71$, $\varphi_\mathrm{b} = 0.95$). The color red is used for concepts related to the top boundary layer, while blue is used for the bottom one. On the first plot, from left to right, the complete temperature profile is shown in black, while boundary layers are emphasized with shaded regions. The values of φ_∞ used for the definition of the boundary layers are indicated with vertical dashed lines. In the second and third plots, the top and bottom boundary layers are shown in detail, with circles at the location of grid points.

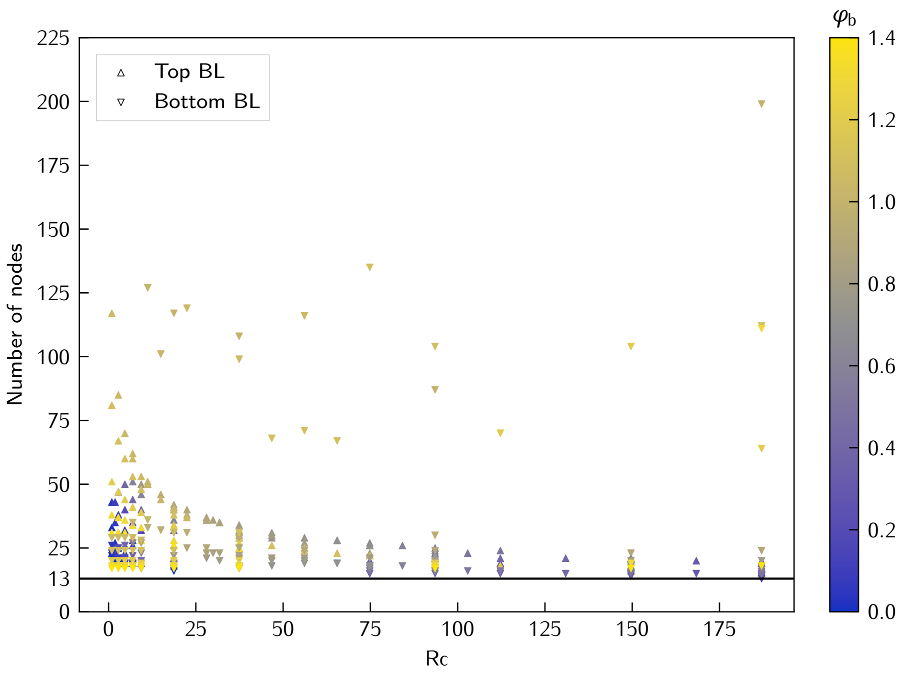

Fig. S7. Number of grid points in the top and bottom boundary layer for each two-dimensional simulation. The color scale shows the value of the bottom temperature φ_b, while Rc is shown in the horizontal axis. A horizontal black line indicates the minimum number of grid points for a boundary layer in our dataset.

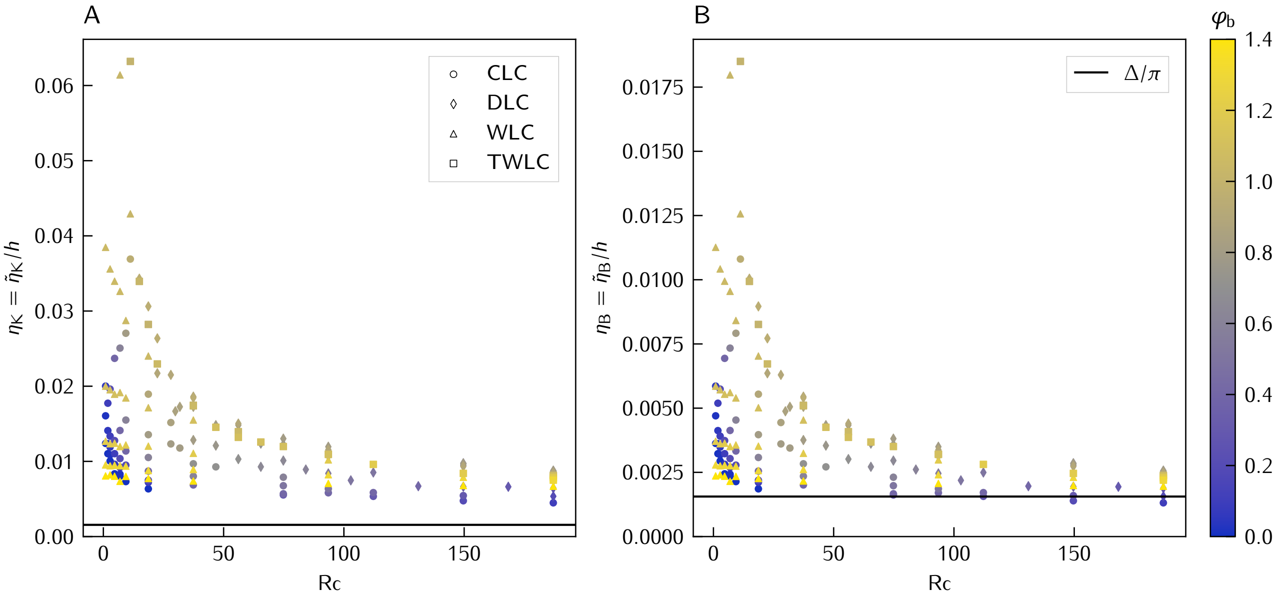

Fig. S8. (*A*) Kolmogorov length-scale and (*B*) Batchelor length-scale (Eq. (31)) as function of Rc (horizontal axis) and φ_b (colorbar) for each convective regime; horizontal continuous line denotes Δ/π (Grotzbach criterion, Eq. 40).

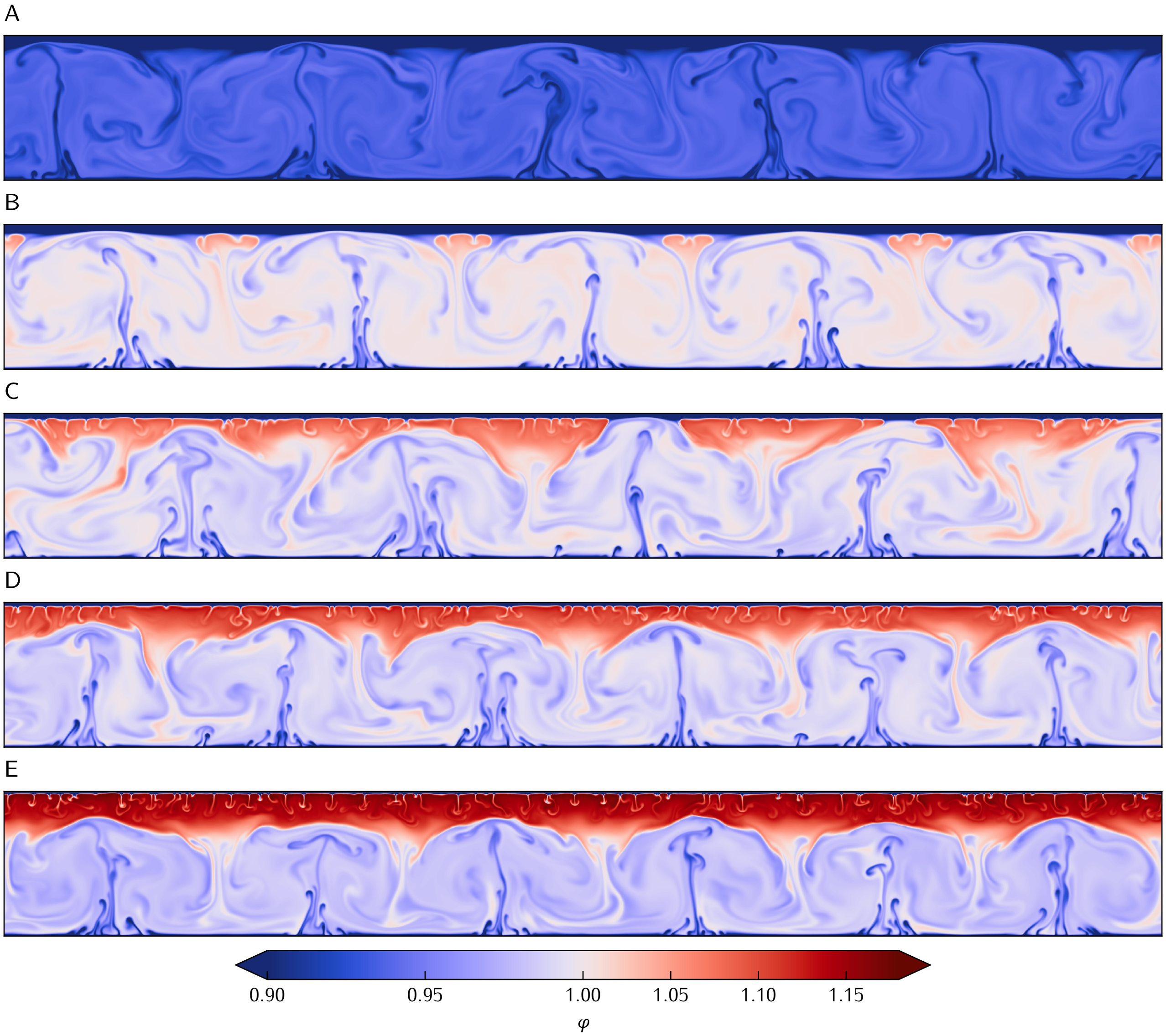

Fig. S9. Example of the transition from Cold Layer Convection (CLC) to Double Layer Convection (DLC) for $\varphi_{\mathrm{b}} = 0.8$ and increasing Rc from 31.8 to 74.8 across panels (*A*)–(*E*), respectively. The colorbar indicates the dimensionless temperature field φ at representative time snapshots from 2D numerical simulations, during the quasi-steady state. (*A*) CLC regime; (*B*) and (*C*) DLC regime with localized convective structures; (*D*) and (*E*) DLC regime with fully developed top convective layer.

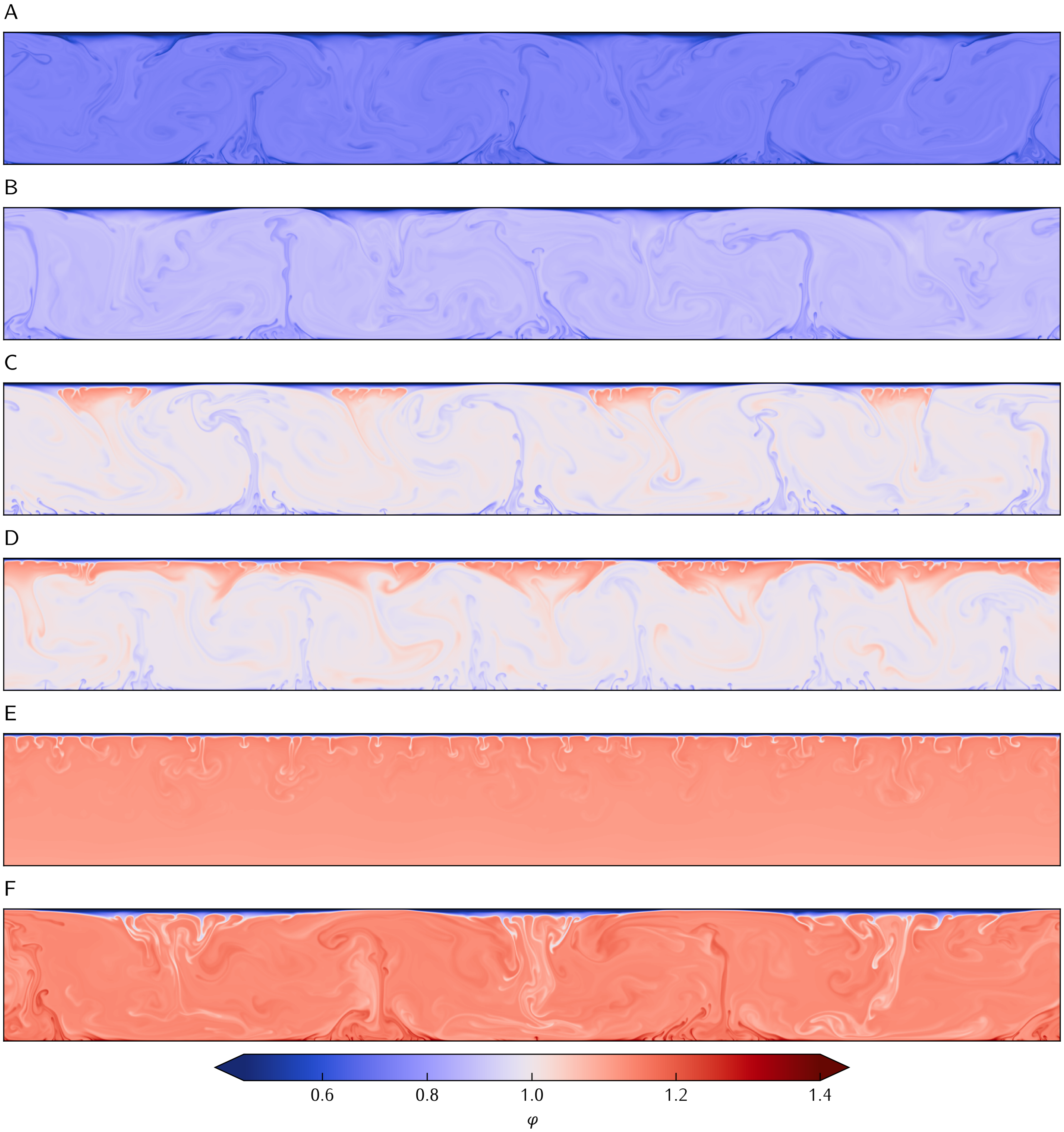

Fig. S10. Examples of different convective dynamics transferring approximately the same water–ice heat flux $|\bar{\Phi}_{\rm i}| \approx 48$: (*A*–*B*) Cold Layer Convection (CLC); (*C*) Double Layer Convection (DLC), partially developed; (*D*) DLC, fully developed; (*E*) Top-Warm Layer Convection (TWLC); (*F*) Warm Layer Convection (WLC). The colorbar indicates the dimensionless temperature field φ at representative time snapshots during the quasi-steady state. The controlling parameters for each case are (*A*) $\mathrm{Rc} = 93.53$, $\varphi_{\rm b} = 0.4$; (*B*) $\mathrm{Rc} = 93.53$, $\varphi_{\rm b} = 0.5$; (*C*) $\mathrm{Rc} = 93.53$, $\varphi_{\rm b} = 0.55$; (*D*) $\mathrm{Rc} = 74.82$, $\varphi_{\rm b} = 0.7$; (*E*) $\mathrm{Rc} = 46.76$, $\varphi_{\rm b} = 1.1$; (*F*) $\mathrm{Rc} = 18.71$, $\varphi_{\rm b} = 1.4$.

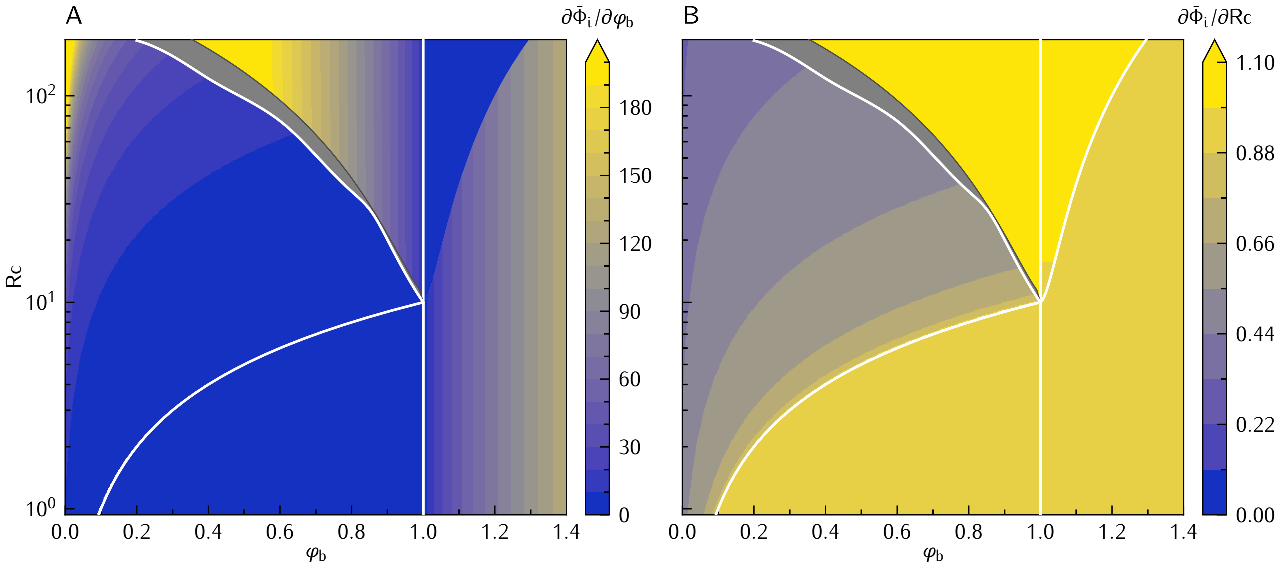

Fig. S11. Variability of water-ice heat flux $|\bar{\Phi}_\mathrm{i}|$ with respect (A) bottom temperature φ_b and (B) radiative-conductive number Rc. White lines denote the regime's boundaries.

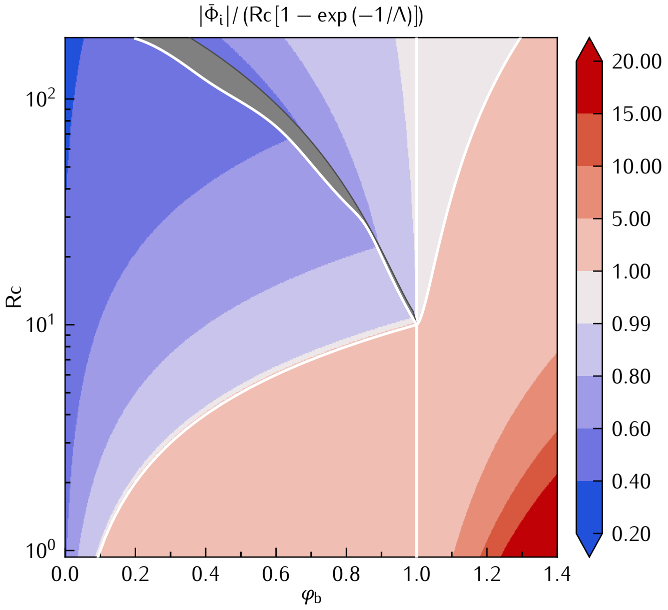

Fig. S12. Ratio between water-ice heat flux $|\overline{\Phi}_{\rm i}|$ and volumetric solar radiation heating rate $\mathrm{Rc}[1-\exp(-1/\Lambda)]$ as a function of the under-ice water body temperature $\varphi_{\rm b}$ and the radiative-conductive number Rc.

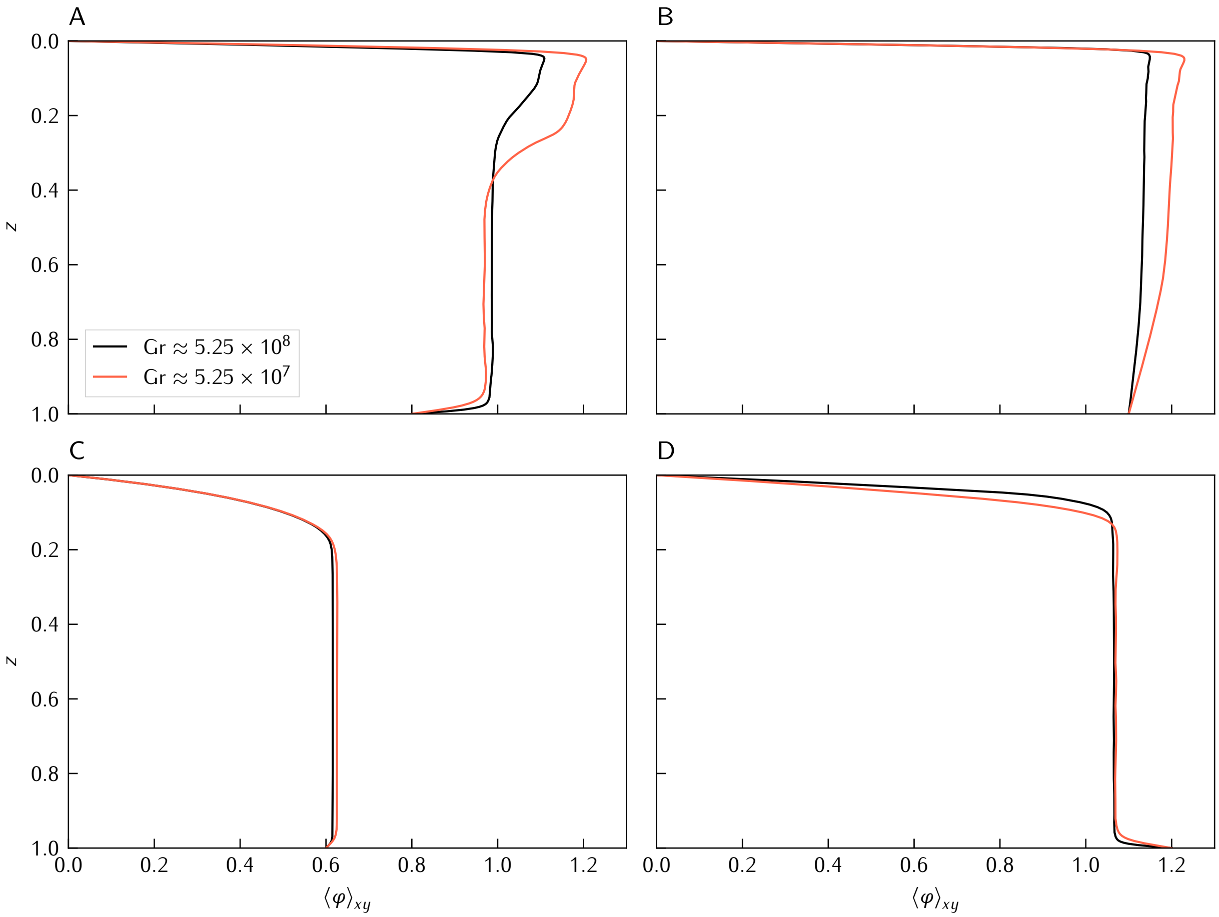

Fig. S13. Comparison of temperature profiles for two Grashof numbers, $\mathrm{Gr} \approx 5.25 \times 10^8$ (black) and $\mathrm{Gr} \approx 5.25 \times 10^7$ (red). To cover the different convective regimes, the following conditions are considered: (A) DLC, $\mathrm{Rc} \approx 56.12$, $\varphi_\mathrm{b} = 0.8$; (B) TWLC, $\mathrm{Rc} \approx 56.12$, $\varphi_\mathrm{b} = 1.1$; (C) CLC, $\mathrm{Rc} \approx 9.35$, $\varphi_\mathrm{b} = 0.6$; (D) WLC, $\mathrm{Rc} \approx 9.35$, $\varphi_\mathrm{b} = 1.2$.

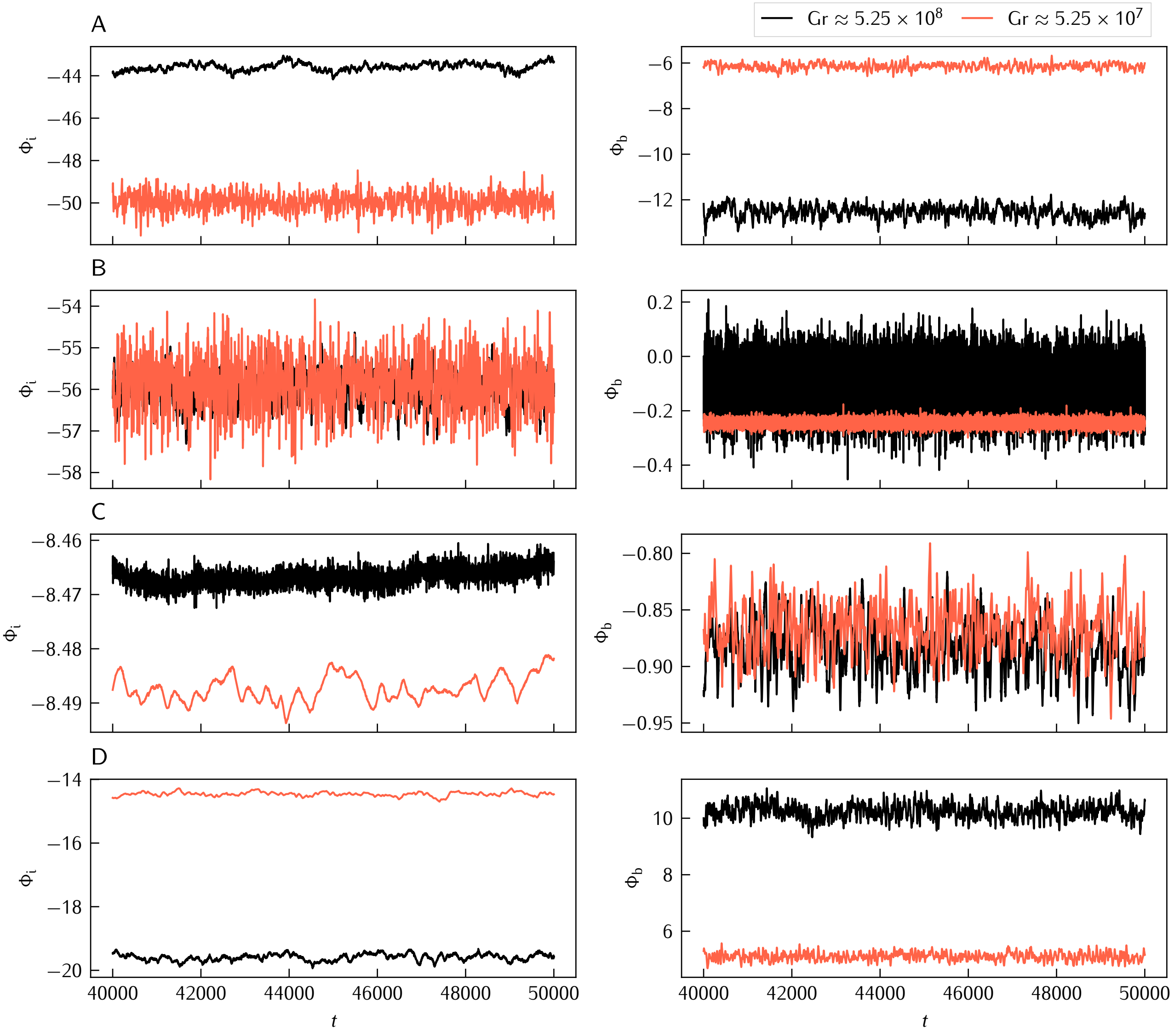

Fig. S14. Comparison of top and bottom heat fluxes, $\Phi_{\rm i}$ and $\Phi_{\rm b}$, for two Grashof numbers, $\mathrm{Gr} \approx 5.25 \times 10^8$ (black) and $\mathrm{Gr} \approx 5.25 \times 10^7$ (red). To cover the different convective regimes, the following conditions are considered: (A) DLC, $\mathrm{Rc} \approx 56.12$, $\varphi_{\rm b} = 0.8$; (B) TWLC, $\mathrm{Rc} \approx 56.12$, $\varphi_{\rm b} = 1.1$; (C) CLC, $\mathrm{Rc} \approx 9.35$, $\varphi_{\rm b} = 0.6$; (D) WLC, $\mathrm{Rc} \approx 9.35$, $\varphi_{\rm b} = 1.2$.

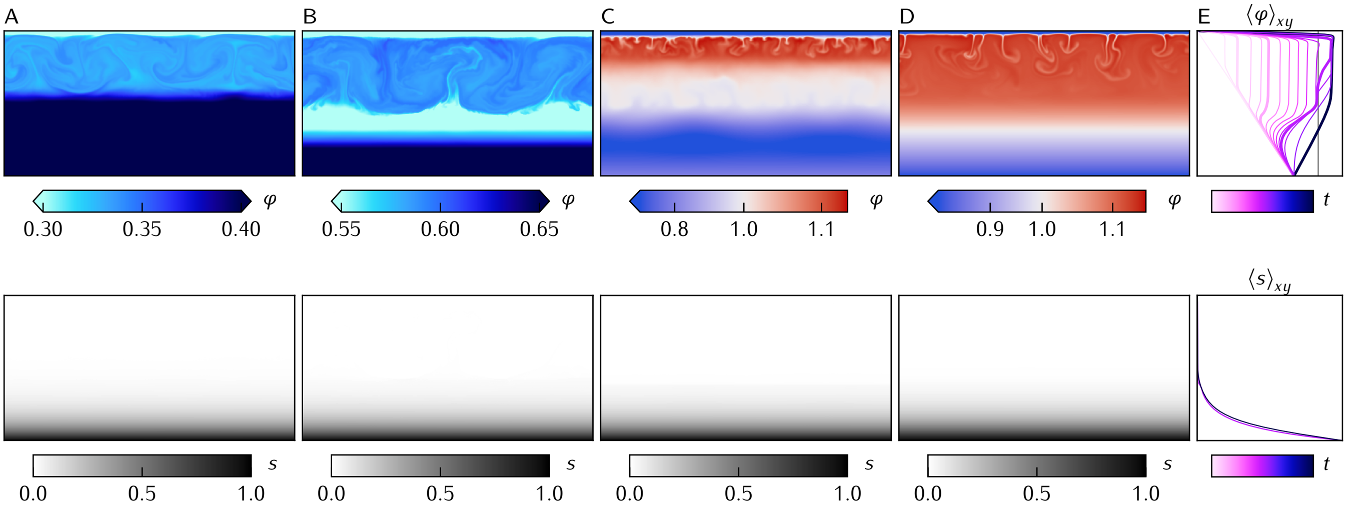

Fig. S15. Transient simulation including the effect of salinity, with conditions $\mathrm{Rc} \approx 46.76$ and $\varphi_\mathrm{b} = 0.8$. From (A) to (D), dimensionless temperature (top) and normalized salinity (bottom) fields are shown for sequential instants of time. (E) shows the time evolution of dimensionless temperature (top) and normalized salinity (bottom) profiles.

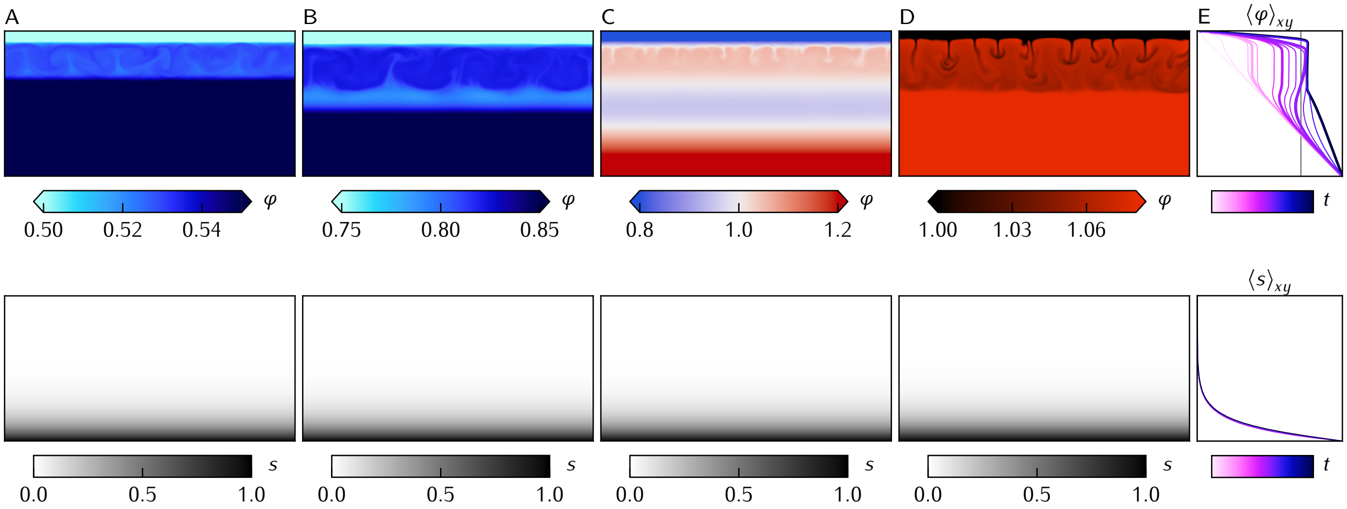

Fig. S16. Transient simulation including the effect of salinity, with conditions $\mathrm{Rc} \approx 22.44$ and $\varphi_\mathrm{b} = 1.4$. From (A) to (D), dimensionless temperature (top) and normalized salinity (bottom) fields are shown for sequential instants of time. (E) shows the time evolution of dimensionless temperature (top) and normalized salinity (bottom) profiles.

Table S1. Parameters for 3D numerical simulations

Regime	Pr	Gr	Λ	φ_{b}	Rc	Aspect ratio: $L_x : L_y : L_z$	$n_x \times n_y \times n_z$
**CLC**	11.67	5.25×10^8	0.1	0.75	9.35	$3:3:1$	$384 \times 384 \times 256$
**DLC**	11.67	5.67×10^8	0.1	0.95	18.71	$3:3:1$	$384 \times 384 \times 256$
**WLC**	11.67	5.67×10^8	0.1	1.06	5.61	$4:4:1$	$384 \times 384 \times 256$
**TWLC**	11.67	5.67×10^8	0.1	1.0	18.71	$1.7:1.7:1$	$384 \times 384 \times 256$

Supplementary Videos

Movie S1. Cold-Layer Convection (CLC): Video showing the three-dimensional convective dynamics associated with the CLC regime at quasi-steady state; colormap denotes the dimensionless temperature fluctuations '$\varphi - \langle\varphi\rangle_{xy}$', highlighting plumes, cell structures, and the single convective mixing layers. Numerical parameters: Rc ≈ 9.35 and $\varphi_\mathrm{b} = 0.75$.

Movie S2. Double-Layer Convection (DLC): Video showing the three-dimensional convective dynamics associated with the DLC regime at quasi-steady state; colormap denotes the dimensionless temperature fluctuations '$\varphi - \langle\varphi\rangle_{xy}$', highlighting plumes, cell structures, and the two convective mixing layers. Physical parameters: Rc ≈ 18.71 and $\varphi_\mathrm{b} = 0.95$.

Movie S3. Warm-Layer Convection (WLC): Video showing the three-dimensional convective dynamics associated with the WLC regime at quasi-steady state; colormap denotes the dimensionless temperature fluctuations '$\varphi - \langle\varphi\rangle_{xy}$', highlighting plumes, cell structures, and the single warm convective mixing layer, centered in the deeper region. Physical parameters: Rc ≈ 5.61 and $\varphi_\mathrm{b} = 1.06$.

Movie S4. Top Warm-Layer Convection (TWLC): Video showing the three-dimensional convective dynamics associated with the TWLC regime at quasi-steady state; colormap denotes the dimensionless temperature fluctuations '$\varphi - \langle\varphi\rangle_{xy}$', highlighting plumes, cell structures, and the single convective mixing layer in the upper region above a stably stratified deeper region. Physical parameters: Rc ≈ 18.71 and $\varphi_\mathrm{b} = 1.00$.

Movie S5. Transient convective dynamics: Video showing the Physical parameters: Rc $= 46.8$ and $\varphi_\mathrm{b} = 0.8$.

SI Dataset S1 (dataset_numerical_simulations.csv)
Table reporting dimensionless numbers, regimes, and numerical parameters.

SI Dataset S2 (Numerical-Results)
Dataset to evaluate the conclusions of this paper is provided in link.

References

1. W Wagner, A Pruß, The iapws formulation 1995 for the thermodynamic properties of ordinary water substance for general and scientific use. *J. Phys. Chem. Ref. Data* **31**, 387–535 (2002).
2. J Gómez, Python implementation of standard from iapws (https://github.com/jjgomera/iapws) (2021).
3. A Oberbeck, Über die Wärmeleitung der Flüssigkeiten bei Berücksichtigung der Strömungen infolge von Temperaturdifferenzen. *Ann. Phys. Chem.* **243**, 271–292 (1879).
4. J Boussinesq, *Théorie analytique de la chaleur: mise en harmonie avec la thermodynamique et avec la théorie mécanique de la lumière.* (Gauthier-Villars) Vol. 2, (1903).
5. M Leppäranta, *Freezing of lakes and the evolution of their ice cover.* (Springer), (2015).
6. D Mironov, et al., Radiatively driven convection in ice-covered lakes: Observations, scaling, and a mixed layer model. *J. Geophys. Res. Ocean.* **107**, 7–1 (2002).
7. D Bouffard, et al., Under-ice convection dynamics in a boreal lake. *Inland Waters* **9**, 142–161 (2019).
8. KJ Burns, GM Vasil, JS Oishi, D Lecoanet, BP Brown, Dedalus: A flexible framework for numerical simulations with spectral methods. *Phys. Rev. Res.* **2**, 023068 (2020).
9. D Noto, JA Letelier, HN Ulloa, Plume-scale confinement on thermal convection. *Proc. Natl. Acad. Sci. U.S.A.* **121**, e2403699121 (2024).
10. G Grötzbach, Spatial resolution requirements for direct numerical simulation of the Rayleigh-Bénard convection. *J. Comput. Phys.* **49**, 241–264 (1983).
11. RJ Stevens, R Verzicco, D Lohse, Radial boundary layer structure and Nusselt number in Rayleigh–Bénard convection. *J. Fluid Mech.* **643**, 495–507 (2010).
12. B Gayen, RW Griffiths, GO Hughes, JA Saenz, Energetics of horizontal convection. *J. Fluid Mech.* **716**, R10 (2013).
13. J Leyrer, H Ulloa, J Ortega, J Letelier, Onset of cabbeling instabilities in superconfined two-fluid systems. *Phys. Fluids* **36** (2024).
14. O Shishkina, RJ Stevens, S Grossmann, D Lohse, Boundary layer structure in turbulent thermal convection and its consequences for the required numerical resolution. *New J. Phys.* **12**, 075022 (2010).
15. HN Ulloa, JA Letelier, Energetics and mixing of thermally driven flows in Hele-Shaw cells. *J. Fluid Mech.* **930**, A16 (2022).
16. P Huo, et al., Strong under-ice heating of Central Asian shallow lakes. *Geophys. Res. Lett.* **52**, e2024GL114501 (2025).
